# Real-Time Machine Learning Model to Predict Cardiac Arrest Within 12 Hours in Critically Ill Patients (DeepiCARS): A Confirmatory Trial and External Validation

**DOI:** 10.3390/diagnostics16213671

**Published:** 2026-11-06

**Authors:** Ukdong Gim, Leerang Lim, Kyung-Jae Cho, Woo Hyun Cho, Hyung-Chul Lee

**Affiliations:** 1 VUNO Inc., Seoul 06541, Republic of Korea; ukdong.gim@vuno.co (U.G.); kcho035@vuno.co (K.-J.C.); 2 Department of Anesthesiology and Pain Medicine, Seoul National University College of Medicine, Seoul National University Hospital, Seoul 03080, Republic of Korea; erange@snu.ac.kr (L.L.); 3 Division of Allergy, Pulmonary and Critical Care Medicine, Department of Internal Medicine, Pusan National University Yangsan Hospital, Pusan National University School of Medicine, Yangsan 50612, Republic of Korea; 4 Transplant Research Center, Research Institute for Convergence of Biomedical Science and Technology, Pusan National University Yangsan Hospital, Yangsan 50612, Republic of Korea; 5 Healthcare AI Research Institute, Seoul National University Hospital, Seoul 03080, Republic of Korea

**Keywords:** cardiac arrest, intensive care unit, early warning score, machine learning, deep learning, external validation, alarm fatigue, clinical decision support

## Abstract

**Background/Objectives**: Continuous prediction of cardiac arrest in the intensive care unit (ICU) must be judged by considering alarm burden as well as discrimination. We evaluated DeepiCARS (iCARS), an approved recurrent-network and gradient-boosting ensemble, developed at one centre and temporally validated there, predicting in-ICU cardiac arrest within 12 h. **Methods**: The unchanged model was assessed in a retrospective 1:1 matched confirmatory trial at that centre (214 adults, 107 with arrest), which assigned no intervention and required no registration, and in 30,487 adult ICU admissions at an independent Korean tertiary centre (2010–2021; 2782 with an in-window arrest: 653 with cardiopulmonary resuscitation [CPR] and 2129 deaths without CPR), against seven conventional scores, per subject in the trial and per prediction point externally, on discrimination, sensitivity at matched alarm burden, lead time and calibration. **Results**: The area under the receiver operating characteristic curve (AUROC) with the extended input (vital signs, Glasgow Coma Scale, age, sex and 21 laboratory values) was 0.9428 (95% confidence interval 0.9102–0.9753) in the trial and 0.9587 (0.9548–0.9626) externally, and with the core (non-laboratory) input 0.9103 (0.8716–0.9490) and 0.9458 (0.9415–0.9500), above every comparator. For arrests with CPR, the external AUROC was 0.8697 (0.8538–0.8854) with the extended input and 0.8425 (0.8264–0.8591) with the core input, above the National Early Warning Score 2 (NEWS2), 0.7157 (0.6987–0.7319), and the Sequential Organ Failure Assessment (SOFA), 0.7424 (0.7205–0.7631). At no greater alarm burden than NEWS2 ≥ 7 and SOFA ≥ 11, iCARS with the extended input detected 37.17 (35.75–38.65) and 30.66 (28.76–32.65) percentage points more of the points preceding an arrest, with median lead times within the 12 h window of 11.42 h and 11.50 h; the score was already above threshold when the window opened in 53.8% and 58.3% of admissions with an arrest, and first crossed it a median of 72.42 h and 80.84 h before the arrest. **Conclusions**: Evaluated unchanged, the approved model retained high discrimination externally, including for arrests with CPR, and detected more of the points preceding an arrest than conventional scores at no greater alarm burden. No operating threshold is proposed.

## 1. Introduction

In-hospital cardiac arrest (IHCA) affects approximately 290,000 adults each year in the United States, and fewer than one in four survive to discharge [1,2]. More than half of these events occur in the intensive care unit (ICU) [3]. Cardiac arrest in the ICU (ICU-CA) complicates 2–3% of ICU admissions [4,5,6], and its prognosis is worse than that of IHCA elsewhere in the hospital: survival to discharge is 15–21% [3,4,7] and one-year survival is below 40% [8]. At the time of arrest, 57–70% of ICU patients are already receiving vasopressors and 63–90% are mechanically ventilated [6,9]; survival halves in patients on vasopressors (9.3% versus 21.2%) and falls further with mechanical ventilation [7], and the burden of organ dysfunction before the arrest is the factor most consistently associated with outcome afterwards [5,9]. Because physiological reserve is already largely exhausted at the time of arrest, improvements in resuscitation can add little, and current guidelines accordingly recommend early warning systems and rapid-response teams to recognise deterioration and prevent in-hospital arrest [10,11,12].

Conditions in the ICU are, in principle, favourable for anticipating cardiac arrest. Every patient is continuously monitored, and nurse staffing is the highest in the hospital [13,14]. Most ICU-CAs are not sudden arrhythmic events: the initial rhythm is non-shockable in 77–90% of ICU cases [6,9] and in about three quarters of in-hospital arrests overall [15], reflecting progressive hypoxic, metabolic or circulatory decline rather than a primary electrical event [2,12]; a precipitating condition can be identified in 79% of cases [5]; and vital-sign abnormalities are already present hours before the arrest [16,17,18]. When ICU-CAs are reviewed by expert panels, more than half contain a potentially preventable element, with delayed response to clinical deterioration, the most frequently identified contributor [19]; survival is higher when the cause of arrest was recognised beforehand [20] and lower at night, when surveillance is thinnest [21]. ICU teams therefore generally recognise that a patient is deteriorating, but cannot reliably identify which deteriorating patient will arrest within the next several hours.

The instruments charted at the ICU bedside were not designed for this task. The severity-of-illness scores—the Acute Physiology and Chronic Health Evaluation II (APACHE-II), the Simplified Acute Physiology Score II (SAPS-II) and the Sequential Organ Failure Assessment (SOFA)—were developed to adjust mortality risk across populations or to describe organ dysfunction from admission or daily data, not to signal that an individual patient is about to arrest [22,23,24,25]. The National Early Warning Score 2 (NEWS2), the aggregate trigger most widely used for deterioration, was derived from intermittently charted ward observations and discriminates cardiac arrest only moderately even in the ward setting for which it was developed [26,27]. Three features limit it: the score is updated only at the frequency of nursing observation rounds; each parameter is weighted independently of the others, so that neither the trajectory of a variable nor the interaction between organ systems contributes; and clinically important deterioration before arrest can fail to trigger it at all [28]. Models built on hospital-wide inpatient data also transfer poorly to intensive care: arrest models that discriminated well in the wards of one hospital fell to an area under the curve of 0.58–0.65 in the intensive care subgroup of the same data [29]. Lowering a score’s threshold increases sensitivity at the cost of a disproportionate rise in alerts [30,31], in an environment in which alarm volume is already high. ICU patients generate about 187 audible monitor alarms per bed per day and 89% of arrhythmia alarms are false [32]; only 15% of monitor alarms are clinically relevant [33], and across all ICU device alarms fewer than 6% lead to a physician being called [34]; the resulting alarm fatigue is a recognised patient-safety hazard [35,36], and exposure to non-actionable alarms measurably slows the response to the next one [37]. The ICU therefore carries a high background rate of non-actionable alarms while lacking a reliable signal for impending cardiac arrest. A predictor added to this environment must be judged by its sensitivity at a given alarm rate rather than by discrimination alone [38]: an ICU early warning system has to reach high sensitivity at a low alarm rate.

Machine learning models that read the electronic health record and re-estimate risk whenever new data arrive can meet both requirements, and in critical care they have been developed for circulatory failure [39], acute kidney injury [40], sepsis [41], short-term and dynamically updated mortality [42,43] and readmission after ICU discharge [44]. In the ICU the most frequent target of such models is death [45], and cardiac arrest has received markedly less attention: of the 36 studies in a recent scoping review of dynamic ICU prediction models, 17 targeted mortality and 12 sepsis, whereas only two, both grouped under cardiovascular complications, targeted cardiac arrest [45]. Real-time models for in-ICU cardiac arrest have been reported [46,47], but almost all remain algorithm-level research outputs, retrospective and single-database, often without alarm burden quantified [48,49,50]; the algorithms that have reached clinical use have degraded from in silico evaluation to clinical deployment and have needed alert suppression or threshold reconfiguration to be tolerated [51,52]. Most artificial-intelligence devices authorised by regulators rest on limited evidence [53]; to our knowledge, no model predicting cardiac arrest in the ICU has been carried through the evaluation a stringent regulator demands of a medical device.

We therefore evaluated DeepiCARS (VUNO Inc., Seoul, Republic of Korea; hereafter iCARS), a prognostic machine learning medical device approved by the Korean Ministry of Food and Drug Safety that predicts in-ICU cardiac arrest within the next 12 h from routinely recorded electronic health record data. It generates a new score each time a vital sign or laboratory value is entered, rather than once per nursing observation round, and computes each score from the record accumulated up to that moment rather than from the most recent observation alone. The device is approved in two input configurations: a core input of vital signs, the Glasgow Coma Scale (GCS) components, age and sex, and an extended input that adds 21 laboratory values. The core input retains the variables of the same manufacturer’s approved general-ward device, DeepCARS, and adds the oxygen saturation and consciousness items routinely charted in intensive care; on the wards, DeepCARS detected arrest with fewer alarms than the Modified Early Warning Score (MEWS) at matched sensitivity [54] and has since been validated prospectively across multiple hospitals [55] and retrospectively at an external hospital [56]. iCARS is in this sense the intensive care counterpart of that ward device: a patient escalated from a general ward can continue to be scored in the ICU on the same monitoring stream, without a separate data-collection pathway.

We evaluated the device unchanged in three layers: internal temporal validation at the development institution, a confirmatory clinical trial with prespecified endpoints, and external validation at an independent institution [57]. Beyond discrimination we quantified alarm burden per bed-day at clinically interpretable comparator cut-offs. We hypothesised that iCARS would discriminate in-ICU cardiac arrest more accurately than the conventional scores and, at equal or higher sensitivity, would generate substantially fewer alarms.

## 2. Materials and Methods

### 2.1. Study Design and Data Sources

The study is reported in accordance with TRIPOD+AI (Transparent Reporting of a multivariable prediction model for Individual Prognosis Or Diagnosis + Artificial Intelligence) [58]. We evaluated iCARS in three layers: internal temporal validation at the development site (Seoul National University Hospital, SNUH; the internal test set), a confirmatory clinical trial at the same institution with prespecified endpoints, reported from the regulatory clinical study report (CSR), and external validation at an independent institution (Pusan National University Yangsan Hospital, PNUYH; the external cohort). Trial records were retrieved in de-identified form through the institution’s clinical data research system (SUPREME 2.0) under the terms of the review board’s approval. The model was developed on SNUH data from 1 September 2007 to 31 December 2019; its weights and preprocessing were unchanged throughout, with no retraining or re-tuning. The main text reports the confirmatory trial and the external cohort; the internal test set (8178 ICU admissions; Section 2.2) is in the Supplementary Materials (Figures S1C and S3, Tables S6, S9, S10, S13 and S15). The three layers differ in design, so they are never pooled and their discrimination values are never compared with each other.

### 2.2. Participants

The confirmatory trial was a retrospective 1:1 matched case–control study of 214 subjects (107 cases, 107 controls) at SNUH, using previously collected source records from November 2021 to July 2023 and assigning no intervention; its eligibility, matching, sample size and prespecified results are transcribed from the CSR and were not recomputed. The protocol fixed the size: 214 subjects gave 91% power at a one-sided 2.5% significance level against an assumed area under the receiver operating characteristic curve (AUROC) of 0.90 with a success threshold of 0.80. Subjects had to be aged 19 years or older, with in-ICU cardiac-arrest status and the trial-specified clinical information—age, sex, the six vital signs, the three GCS components and their measurement times—retrievable over the screening period; laboratory values were collected but were not an eligibility requirement. Patients who had contributed to model development were excluded, so the trial population does not overlap the development set at the patient level; the second exclusion criterion was any record the investigator judged liable to affect the result, for example, through a medical-record error. The CSR gives no count for either criterion. Cases were drawn at random from the eligible arrest admissions, and for each, a control was drawn at random from the non-arrest admissions matching it based on sex, ten-year age band and ICU type, by a staff member independent of the investigators. For a control, the window was the 12 consecutive hours ending at a time point selected at random during the ICU stay.

The trial prespecified one co-primary efficacy endpoint for each input configuration: the lowest AUROC across the evaluated horizons, with efficacy to be declared if, for both configurations, the lower limit of the 95% confidence interval (CI) at that horizon reached 0.80. Sensitivity, specificity, the mean interval from first above-threshold prediction to event and subgroup analyses by sex, age and ICU type were prespecified secondary endpoints. Predictive values, accuracy and the calibration plot were added after protocol approval and are identified as unplanned in the CSR.

**Figure 1:**
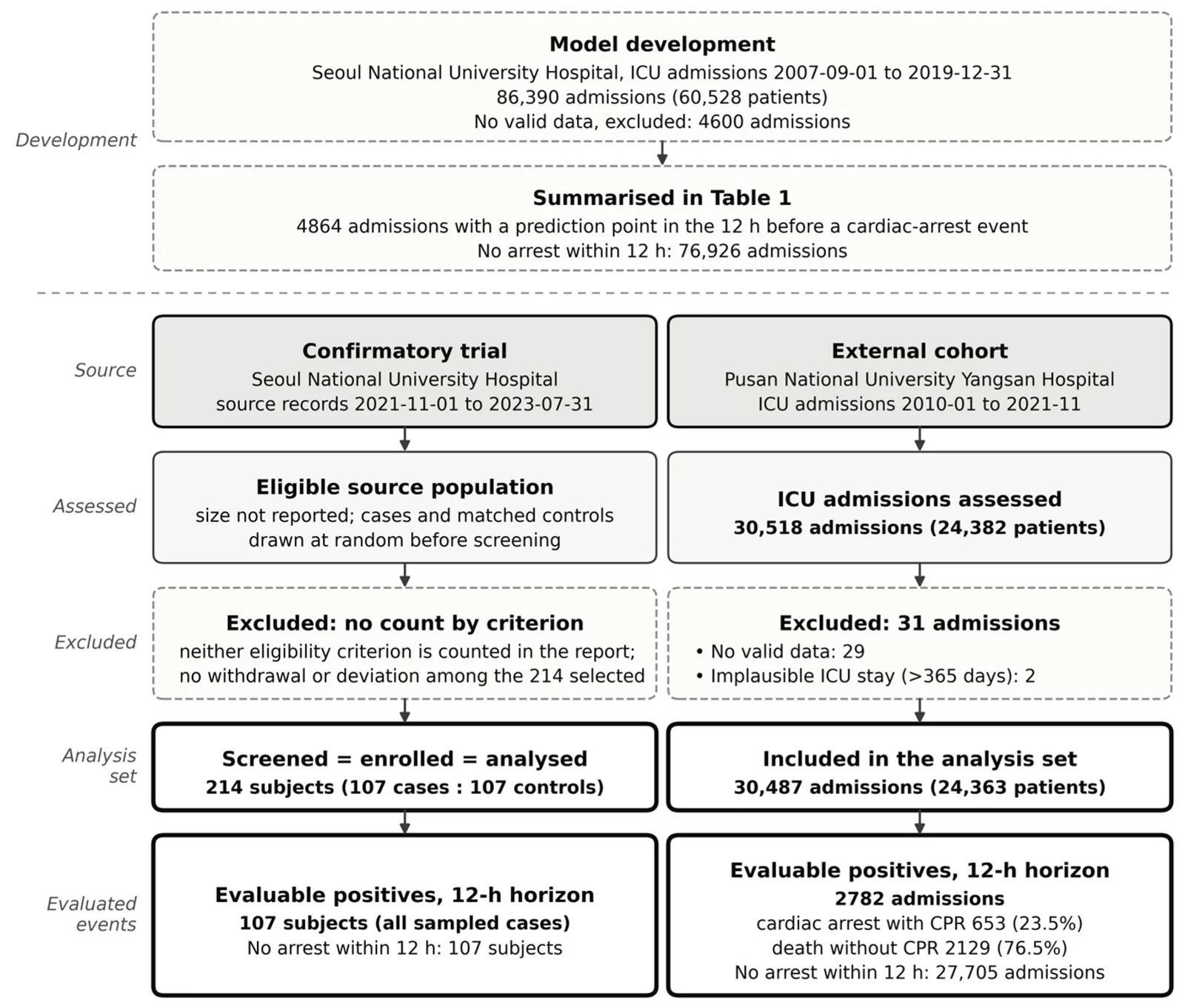
Study flow. The development cohort contributed no evaluation and is drawn outside the staged grid; patients admitted in its period are excluded from the internal test set (Figure S3). Event types, cardiac arrest with cardiopulmonary resuscitation (CPR) and death without CPR, are shown for the external cohort. ICU = intensive care unit.

The external cohort comprised all ICU admissions at PNUYH from January 2010 to November 2021: 30,487 admissions from 24,363 patients after exclusions. The internal test set comprised all SNUH ICU admissions from January 2020 to August 2021, excluding at the patient level every patient with an ICU admission in the development period. All three are adult ICU cohorts; the numbers excluded are in Figure 1 for the external cohort and Figure S3 for the internal test set. Neither cohort had a sample-size calculation; their confidence intervals show the precision obtained.

### 2.3. Prediction Model

**Figure 2:**
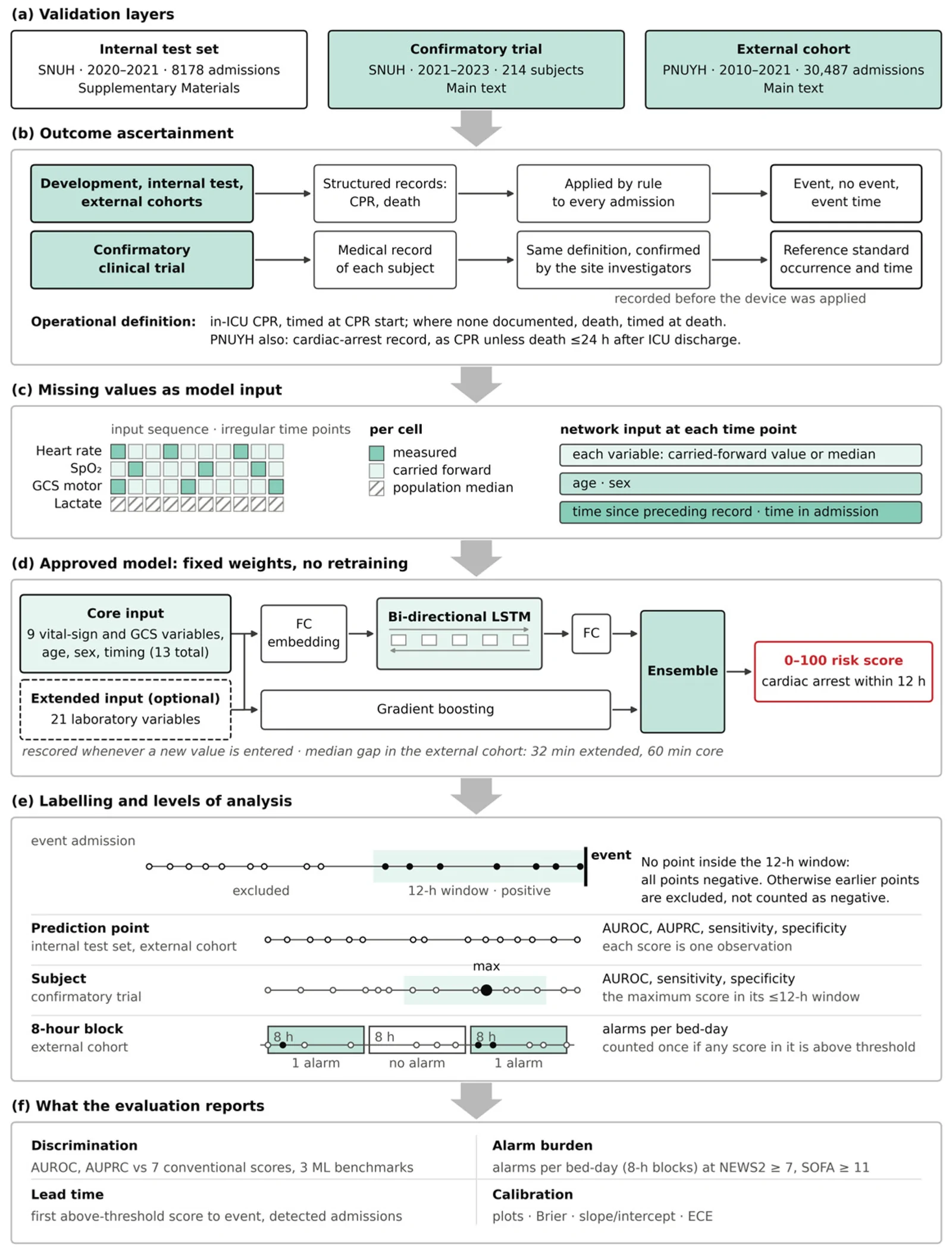
Study design, outcome ascertainment, model and levels of analysis. (**a**) The three validation layers and their data sources. (**b**) Outcome ascertainment—by rule from structured records in the retrospective cohorts, and in the confirmatory trial by the site investigators before the device was applied. (**c**) What the network receives at each record of the input sequence: each variable’s last value carried forward within the admission, or the population median before its first observation, with age, sex and two timing features—the interval since the preceding record and the time elapsed in the admission. (**d**) The approved engine: a bi-directional long short-term memory network and a gradient-boosting model read the inputs in parallel and are ensembled into the 0–100 score; weights and preprocessing are fixed. (**e**) Labelling, and the level at which each reported quantity is computed. (**f**) The four quantities the evaluation reports. AUPRC = area under the precision–recall curve; AUROC = area under the receiver operating characteristic curve; ECE = expected calibration error; FC = fully connected; GCS = Glasgow Coma Scale; LSTM = long short-term memory; ML = machine learning; NEWS2 = National Early Warning Score 2; PNUYH = Pusan National University Yangsan Hospital; SNUH = Seoul National University Hospital; SOFA = Sequential Organ Failure Assessment; SpO2 = peripheral oxygen saturation.

iCARS rescales its predicted probability to a 0–100 risk score, updated whenever new input becomes available (Figure 2). The model was trained on an outcome-balanced sample, and the score is reported on that scale, in which events and non-events carry equal weight. The trial used the device’s output as is; outside the trial, the output was additionally calibrated on the development data, on this scale and as the absolute risk of arrest within 12 h. The approved engine analyses the input sequence with a bi-directional recurrent neural network built on long short-term memory units together with fully connected layers, ensembled with a gradient-boosting model, so each score is a function of the preceding sequence within that admission, not of the current observation alone. Because cardiac arrest is rare at the prediction-point level, the positive class was over-represented by batch-level sampling during development, and the gradient-boosting component was fitted with class weights. The core input, which the regulatory documentation calls the required input, is 13 features (vital signs including the three GCS components, age, sex and timing features) and it retains the variable set of the manufacturer’s general-ward device; the extended input adds 21 laboratory variables, 34 in total. Each variable enters as its last observation carried forward within the ICU admission, with no limit on how long it may be carried, or as the population median for an input not yet observed in that admission, and the two timing features—the interval since the preceding record and the time elapsed in the admission—convey when each record was made; the sequence of records thus carries the measurement pattern, which in intensive care is itself informative because a variable is measured when a clinician has reason to measure it [39,59]. The extended input therefore tolerates absent laboratory values and can score a unit whose laboratory coverage is partial, whereas the core input carries no laboratory variable. No prediction point was excluded for partial missingness. An unassessable GCS verbal response, recorded as a letter code at SNUH and as 0 at PNUYH, entered the device as missing at both institutions, in keeping with the device’s input range of 1 to 5.

The trial threshold of 66 is the value carried by the final approved protocol. Following the convention fixed in the confirmatory trial, the core-input model scores only prediction points at which at least one of the nine vital-sign or GCS variables was recorded, whereas the extended-input model scores every point; the two configurations are therefore compared only on the points they share.

Preprocessing, quality checking and prediction were carried out by the approved engine as deployed (model VN-C-13), identically for every admission; the authors did not re-implement it. The device requires no user entry: it reads structured record fields automatically and displays the score and its trajectory to the ICU clinical team. All predictors were extracted automatically from structured fields, so the extraction procedure was identical across subgroups, although charting frequency may itself differ between patient groups.

### 2.4. Outcome Definition

In the confirmatory trial, the reference standard was whether an in-ICU cardiac arrest occurred and, where it did, the time of onset. Candidate events were identified from the medical record by the operational definition below, then reviewed and confirmed, with their event times, by the site investigators under the principal investigator, a specialist in the Department of Anesthesiology and Pain Medicine. The confirmed occurrence and onset time, not the event type, were recorded on the case report form before the device was applied, and the investigator applying the device was blinded to them (Figure 2b).

In the development, internal test, and external cohorts, no case-by-case review was possible, so the event was defined operationally from structured records alone, following in-hospital cardiac-arrest reporting conventions [60]: cardiopulmonary resuscitation (CPR) during the ICU stay, timed at CPR start, and, where no in-ICU CPR was documented, death, timed at death. At PNUYH the structured record holds two event types, CPR and cardiac arrest, each with a time only. An event was counted as cardiac arrest with CPR if a CPR record existed, or if a cardiac-arrest record was not followed by death within 24 h of ICU discharge; other cardiac-arrest records were counted as deaths without CPR. Every event was timed at the earliest such record. Table S16 gives the record types and their rates before and after the 2018 Korean legislation on life-sustaining treatment decisions. No do-not-resuscitate-based exclusion of deaths was applied, and deaths following withdrawal of life-sustaining treatment cannot be separated from those counted this way. Outcome ascertainment was blind to the model scores. Observation started at the first valid prediction and ended at the earliest of ICU discharge, death or the event; time after the event was excluded from all denominators.

### 2.5. Outcome Window and Prediction Points

The window is the 12 h before the event in the primary analysis, and correspondingly shorter at the 3, 6 and 9 h horizons of Table S5. Prediction points inside it were positive. The event time and everything after it were excluded. In admissions with at least one positive prediction point, points earlier than the window were also excluded from the discrimination analysis rather than counted as negative. All remaining points were negative, under the same convention for iCARS and every comparator. Alarm analyses used the whole observation period, because alarm burden is a rate per bed-day rather than a label-conditional proportion; restricting the alarm set to the discrimination set would raise the reported alarm-reduction factors rather than lower them (Table S12).

Labelling and observation are confined to the ICU admission, so an event falling more than 12 h after ICU discharge contributes no positive prediction point; such an admission is scored negative throughout, and Table 1 and Figure S3 show how many there are in each cohort.

Every reported value states the level at which it was computed: the external cohort and the internal test set at the prediction-point level, the confirmatory trial at the subject level (each subject’s maximum score within the single ≤12 h case-report-form window the CSR fixes for it), and alarm burden at the 8 h block level. The continuously scored cohorts are not summarised per admission, because a maximum taken over a whole stay counts an admission as detected on any single threshold crossing, so sensitivity defined in that way increases with length of stay.

### 2.6. Comparators

Seven conventional scores served as comparators: four bedside deterioration scores—NEWS2 [26], MEWS [61], the Cardiac Arrest Risk Triage (CART) score [62] and the quick Sequential Organ Failure Assessment (qSOFA) [63,64]—and three severity-of-illness scores, SOFA [24], APACHE-II [22] and SAPS-II [23], with their original item weights [65]. All seven were computed retrospectively from the same records the device reads, each on the convention its own source specification prescribes rather than on a single imputation rule: NEWS2 and MEWS from the parameters observed at each prediction point and, where a parameter is unavailable, from the remainder, following Royal College of Physicians guidance that such a score “should still be calculated and documented as incomplete” [66]; qSOFA, CART and the three severity scores from the most recently available component values within the ICU admission. Where the trial’s 31-item case report form could not support an instrument in full, the score is marked with an m prefix, and severity-score discrimination is not compared between the two cohorts.

SOFA was the primary severity comparator. Alarm-burden comparisons are anchored at NEWS2 ≥ 7 and SOFA ≥ 11, with NEWS2 ≥ 6 and SOFA ≥ 10 as secondary anchors—clinically interpretable comparator cut-offs rather than thresholds selected for the sensitivity they reach.

Three machine learning models—logistic regression, random forest and gradient boosting—were fitted for this analysis as benchmarks, unlike the approved device, which was evaluated unchanged. They were trained on the development set and evaluated in the internal test set and the external cohort, on the device’s own features, carried forward as for the device and otherwise set to the development-set median, with an indicator for each variable that no value had yet been observed, standardised for logistic regression and unscaled for the tree-based models; no resampling or class weighting was applied.

### 2.7. Alarm Burden

Alarm burden was the mean alarm count per bed-day (MACPD) [42], alarm units divided by observed bed-days, one bed-day being one day of one occupied ICU bed, so MACPD is bed-day-weighted rather than an average of per-admission rates. The primary alarm unit was the 8 h block, the length of a nursing shift, though not aligned to institutional shift boundaries: each admission’s observation period was divided into consecutive blocks, and a block counted as one alarm if the highest score inside it reached the threshold. The number needed to evaluate (NNE), the reciprocal of the positive predictive value (PPV), was computed at the prediction-point level, and all main-text alarm results come from the external cohort. These datasets do not record whether an installation mutes repeat alerts, so no device alerting policy is assumed.

Each comparator cut-off fixes an alarm budget—how often, per bed-day, an 8 h block contains an alarm—and iCARS is held to that budget and compared on the share of pre-event observations it flags (Section 3.3); Table S3 extends that reading to all seven comparators. Alarm burden itself is compared the other way round, at matched sensitivity (Section 3.4, Table S1), and every instrument is evaluated on the same 8 h block cadence. iCARS is matched to each comparator cut-off by the lowest iCARS threshold on the 0–100 sweep grid whose alarm burden does not exceed that of the comparator under both the 8 h block and the raw definition, and the difference in sensitivity is reported; for the reciprocal reading, it is matched by the highest threshold whose sensitivity is at least that of the comparator. Only the 101 integer values of the 0–100 score are available as thresholds, so neither match is exact and the matched threshold is the conservative one on the quantity held fixed; for the equal-sensitivity reading, the match on the continuous probability output, which is exact and does not depend on the calibration of Section 2.3, is reported alongside. Both anchor tiers are reported for both input configurations (Table S1). The matched thresholds are computed quantities rather than settings we propose, and the trial threshold of 66 is reported only for the trial’s endpoints specified at it (Section 3.3). Sensitivity, specificity, predictive value and alarm burden from score 50 upward are in Table S2.

### 2.8. Statistical Analysis

Discrimination was summarised by the AUROC with 95% confidence intervals from patient-level clustered bootstrap (2000 resamples); DeLong [67] statistics were computed as a cross-check and are not tabulated. Comparisons with iCARS used paired bootstrap on common prediction points within the same input mode, and the *p* value reported as “≤0.001” when no resampled difference crossed zero. The area under the precision–recall curve (AUPRC) was computed only where the observed prevalence is real, and is reported for the external cohort (Table S5). Outside the trial, iCARS discrimination was computed from the model’s probability output and for each comparator from its own integer scale, so every instrument is evaluated at its native resolution. For the confirmatory trial, only the iCARS point estimates and intervals are CSR transcriptions; all comparator intervals, differences and *p* values there were computed by us with the same subject-level clustered bootstrap applied to the CSR-reproducing pipeline scores. Baseline differences in Table 1 were tested by a prediction-point permutation test, with the event label permuted at the admission level so that repeated measurements within an admission move together.

Operating characteristics were computed at the prediction-point level for the external cohort, at the thresholds matched to the comparator anchors; the trial’s endpoints at threshold 66 are cited from the CSR. Alarm lead time was the interval from the first above-threshold prediction to the event among positive admissions in which an alarm occurred, reported as a median with interquartile range (IQR). Subgroups of the 12 h AUROC were sex, age (<65 versus ≥65 years) and ICU type. Calibration used plots, the observed-to-expected ratio, the Brier score, the calibration slope and intercept (the latter with the slope fixed at 1) and the expected calibration error—the mean absolute difference between predicted and observed risk over twenty equal-count bins of predicted risk—on the absolute 12 h risk of Section 2.3 in the discrimination set; in the confirmatory trial, whose 1:1 design fixes the observed event fraction at 50%, it used the device’s output, with eight equal-count bins because its tied scores allow no more. Intervals cover discrimination, operating characteristics, alarm burden and its ratios, lead times and calibration: Clopper–Pearson exact for the trial’s operating characteristics, a bootstrap resampling cases and controls separately for its expected calibration error, and otherwise the patient-level clustered bootstrap (500 resamples for calibration), each ratio formed within the resample; the trial’s transcribed Brier scores and lead times are point estimates (Table S9, Section 3.5). In-hospital cardiac-arrest incidence and survival differ by age, sex and socioeconomic position, and an automated early warning system can inherit such differences through the charting frequency of the observations it consumes. Fairness was therefore assessed descriptively and could not be enforced on a fixed approved device: both cohorts are Korean tertiary centre ICU populations, so performance is reported by sex, age band and ICU category, while ethnicity, insurance status and socioeconomic position could not be examined. No class-imbalance method was applied in this evaluation; the handling used during development is described in Section 2.3. Analyses were performed in Python 3.12 with pandas 2.2 and scikit-learn 1.5. Claude Code (version 2.1; Anthropic, San Francisco, CA, USA) was used to assist with verification of the analysis code and preparation of figures. All analytical decisions, interpretation of results and conclusions were made and verified by the authors.

### 2.9. Ethics and Registration

Three institutional review board determinations from the two participating hospitals cover the study, and informed consent was waived under all three because only previously collected records were used. The confirmatory trial was additionally approved by the Ministry of Food and Drug Safety before it began and conducted under Korean Good Clinical Practice, International Council for Harmonisation (ICH) guidance and the Declaration of Helsinki. Assigning no intervention prospectively, it falls outside the International Committee of Medical Journal Editors (ICMJE) registration requirement and was not registered. Board numbers are in the Institutional Review Board Statement.

## 3. Results

### 3.1. Cohort Flow and Characteristics

The development period, September 2007 to December 2019, comprised 86,390 SNUH ICU admissions (60,528 patients); after 4600 admissions without valid data were excluded as in the other cohorts (Figure 1), 81,790 (57,654 patients) remained, of which 4864 had at least one prediction point inside the 12 h before a cardiac-arrest event. Of 30,518 admissions (24,382 patients) screened at PNUYH between January 2010 and November 2021, 30,487 (24,363 patients) entered the external cohort (Figure 1), of which 2782 with the extended input and 2770 with the core input were positive on that definition. The confirmatory trial comprised 214 subjects in 107 matched pairs.

Baseline characteristics of all three cohorts, including the distribution of the model’s own input variables by outcome group, are in Table 1. Consciousness and the vital signs were worse in the arrest columns than in the no-arrest columns in all three cohorts, with one exception: externally, respiratory rate was slightly lower in the arrest column, whereas in the trial it was higher. The motor GCS component was 2.54 against 5.06 in the trial and 2.69 against 5.28 externally. Laboratory values moved in the same direction where they moved at all—lactate 9.24 against 1.77 mmol/L and 8.74 against 1.75—but less consistently: glucose separated the groups only slightly in either cohort, and the partial pressure of carbon dioxide (pCO2) not at all in the trial. Native recording was very uneven across input blocks: externally, 64.0–85.6% of prediction points for the six non-consciousness vital signs, 21.3% for all three components of the GCS as charted—11.9% for the verbal component once range-checked (Table S11)—and under 9% for the 21 laboratory values. Consecutive predictions were a median of 32 min apart with the extended input (IQR 27–60) and 60 min with the core input (IQR 30–60), which is scored only where a vital sign or GCS component was measured.

### 3.2. Discrimination

**Figure 3:**
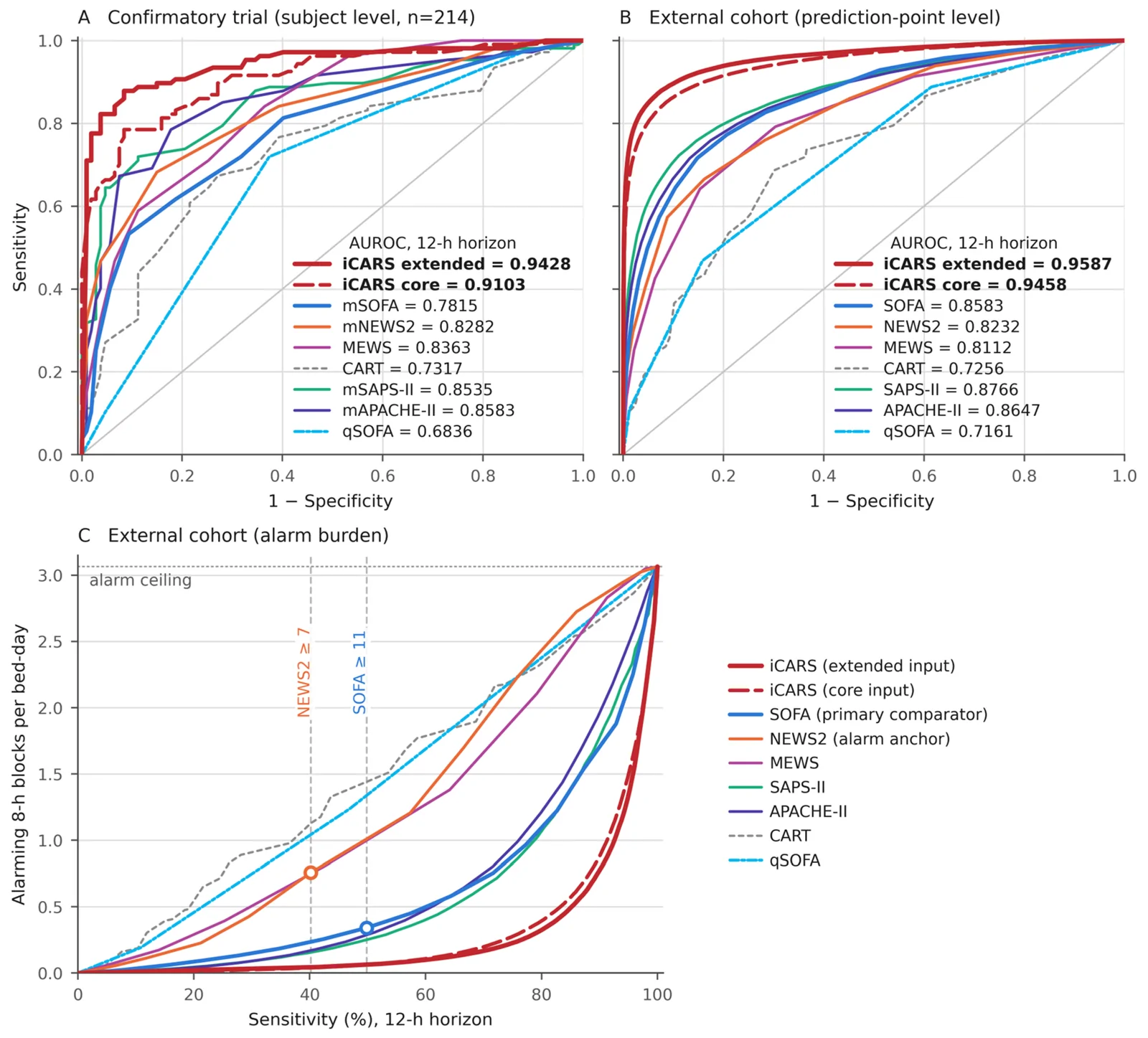
Discrimination and alarm burden. (**A**,**B**) Receiver operating characteristic curves at the 12 h horizon, in the confirmatory trial and in the external cohort. The prefix m marks a comparator reconstructed from the reduced confirmatory-trial item set (Section 2.6); the in-panel legends give point estimates only, with confidence intervals and paired-bootstrap significance in Table 2. (**C**) Mean alarm count per bed-day against sensitivity in the external cohort, with seven comparators traced across their reachable integer cut-offs on the same 8 h block cadence. Dashed lines mark each anchor’s sensitivity, at which open circles mark the two anchor cut-offs, NEWS2 ≥ 7 and SOFA ≥ 11.

In the confirmatory trial, extended-input iCARS discriminated impending cardiac arrest within 12 h with a subject-level AUROC of 0.9428 (95% CI 0.9102–0.9753) and the core-input model 0.9103 (0.8716–0.9490). The co-primary endpoint was the lowest AUROC across horizons, falling at 12 h for the core input and at 9 h for the extended input (0.9312, 0.8954–0.9670); both lower confidence limits exceeded the 0.80 success threshold and the endpoint was met. In the external cohort the prediction-point AUROCs at 12 h were 0.9587 (0.9548–0.9626) and 0.9458 (0.9415–0.9500) (Figure 3B), higher at the 3 h horizon (Table S5), with extended-input AUPRC 0.6922 (0.6597–0.7198). The extended input ranked above the core input in both (trial Δ 0.0325, *p* = 0.0320; external Δ 0.0129, *p* ≤ 0.001).

Extended-input iCARS ranked above every comparator in both cohorts, every difference significant (all bootstrap *p* ≤ 0.001 externally; Table 2, Figure 3A,B). The strongest comparator was full-item SAPS-II at 0.8766 (0.8690–0.8845); the primary comparator SOFA reached 0.8583 (0.8498–0.8665), and every bedside early warning score ranked below all three severity scores, the highest being NEWS2 at 0.8232 (0.8169–0.8295). The reduced-item forms rank differently in the confirmatory trial, where mSOFA falls below the two aggregate early warning scores. In that cohort, the core input also ranked significantly above every comparator (Table 2).

Most arrests were deaths without CPR, 76.5% in the external cohort and 61.1% in the internal test set, while the trial recorded the outcome without its type (Table 1 and Table S13). For arrests with CPR, the extended-input AUROC was 0.8697 (0.8538–0.8854) externally and 0.9031 (0.8775–0.9271) in the internal test set, against 0.9799 (0.9775–0.9823) and 0.9804 (0.9739–0.9861) for deaths without CPR. NEWS2 and SOFA were also lower for arrests with CPR, at 0.7157 (0.6987–0.7319) and 0.7424 (0.7205–0.7631) externally and 0.6967 (0.6602–0.7314) and 0.7471 (0.6971–0.7919) in the internal test set, and iCARS ranked above both for each event type in both cohorts (all *p* ≤ 0.001; Table S13).

Scored as originally defined rather than repeatedly, SAPS-II gave an AUROC of 0.7313 (0.7192–0.7436), far below the repeated form of Table 2 (Table S7).

### 3.3. Operating Characteristics

Headline operating characteristics are reported at the threshold whose alarm burden matches the primary comparator’s reference point, SOFA ≥ 11: iCARS threshold 66 with the extended input and 57 with the core input; the thresholds matched to NEWS2 ≥ 7 are 76 and 62. In the external cohort at threshold 66 the extended input gave a sensitivity of 80.47% (95% CI 79.19–81.60), a specificity of 97.34%, a positive predictive value of 32.74% and a number needed to evaluate of 3.05, at 0.3372 above-threshold 8 h blocks per bed-day against 0.3411 for SOFA ≥ 11; at threshold 57, the core input gave 77.90% (76.63–79.08), 96.17%, 25.15%, 3.98 and 0.3401 (Table S8 and Table S2). At no more than the alarm burden of NEWS2 ≥ 7 and SOFA ≥ 11, whose sensitivities were 40.19% and 49.81%, the extended input detected 37.17 (35.75–38.65) and 30.66 (28.76–32.65) percentage points more (Table S1). In terms of time, with each score held until the next, iCARS was above threshold for 74.0% (95% CI 72.5–75.4) of the observed time in the 12 h before an event at the SOFA ≥ 11 budget (a mean of 8.1 h) against 48.7% (47.0–50.5; 5.3 h) for SOFA ≥ 11, and for 70.4% (68.9–71.9; 7.7 h) at the NEWS2 ≥ 7 budget against 41.3% (40.1–42.5; 4.5 h) for NEWS2 ≥ 7.

The trial threshold of 66 is reported for the trial’s threshold-based secondary endpoints, not as an operating point: 82.24% (73.67–88.96) and 95.33% (89.43–98.47) with the extended input, and 78.50% (69.51–85.86) and 85.05% (76.86–91.20) with the core input. Threshold 66 was derived from the development data and was not tuned on any cohort evaluated here; beyond those endpoints it is not carried into the main-text results.

Prediction-point event prevalence in the external cohort, against which its predictive values are read, is 1.58% (extended) or 1.63% (core).

Counted as negatives, the prediction points earlier than the 12 h window in event admissions lower the AUROC to 0.9375 (NEWS2 0.8039, SOFA 0.8191) and, at the alarm-burden-matched thresholds, specificity and the PPV to 92.83% and 12.56% at threshold 66 against 90.90% and 6.55% for SOFA ≥ 11, and to 94.50% and 15.27% at threshold 76 against 94.02% and 7.92% for NEWS2 ≥ 7 (Table S14).

### 3.4. Alarm Burden at Matched Sensitivity

Alarm burden is quantified in the external cohort only, because the trial’s 1:1 design makes prevalence-dependent measures uninterpretable [68].

At NEWS2 ≥ 7, the aggregate score at which the Royal College of Physicians specifies an emergency response [26], the comparator reached a prediction-point sensitivity of 40.19% at 0.7570 alarms per bed-day, an alarm being an 8 h block whose peak score reached the cut-off. The highest extended-input iCARS threshold reaching that sensitivity was 100, the end of the scale, at which iCARS detected 41.62% at 0.0455 blocks per bed-day, or 16.64-fold fewer (15.53–17.79); matched exactly on the continuous output, the reduction was 17.61-fold (16.44–18.84). At SOFA ≥ 11, high in the score’s range and where reported mortality is correspondingly high [69,70], and the level at which a published critical-care triage protocol sets a ceiling on ongoing treatment [71] rather than triggering a response, the comparator reached 49.81% at 0.3411 blocks per bed-day, against 52.29% at 0.0693 for iCARS at threshold 99, 4.93-fold fewer (4.60–5.28), or 5.48-fold (5.10–5.88) matched exactly (Figure 3C). With the core input, the same two anchors gave 17.48-fold and 5.16-fold, at thresholds 98 and 95, and at the secondary anchors NEWS2 ≥ 6 and SOFA ≥ 10, the extended input generated 12.16-fold and 4.54-fold fewer. Lead times at these equal-sensitivity thresholds are given in Table S8.

### 3.5. Lead Time

Lead time is a timing descriptor conditioned on an alarm having occurred rather than a discrimination metric. At the thresholds matched on alarm burden to NEWS2 ≥ 7 and SOFA ≥ 11, the medians in the external cohort were 11.42 h and 11.50 h with the extended input (thresholds 76 and 66), and 11.27 h and 11.32 h with the core input (62 and 57), all bounded above by the 12 h horizon; at threshold 66 the interquartile range was 7.62–11.78 h (Table S8). Discrimination was retained across the window rather than concentrated in its last hours: in the external cohort the extended-input AUROC was 0.9744 for observations 0–3 h before the event and 0.9451 for those 9–12 h before, and sensitivity at threshold 66 fell from 88.51% to 72.80%, against 38–51% for the two comparator cut-offs throughout (Table S15). In the confirmatory trial the medians transcribed from the CSR were 11.42 h and 11.08 h (means 9.38 h and 8.81 h), at the trial threshold of 66 and on a different observation-window geometry.

### 3.6. Calibration

In the confirmatory trial, whose 1:1 matched design fixes the observed event fraction at 50%, the clinical study report gave Brier scores of 0.0864 with the extended input and 0.1270 with the core input on the device’s output (Figure S1A); on the pipeline scores that reproduce the CSR, the mean predicted values were 49.10% and 55.86% and the expected calibration errors 0.0321 (95% CI 0.0249–0.0849) and 0.0664 (0.0571–0.1358) (Table S9).

Outside the trial, calibration was assessed on the discrimination set (Section 2.5). In the external cohort, the absolute 12 h risk read with the development-data calibration of Section 2.3 averaged 1.48% and 1.25% against observed event rates of 1.58% and 1.63%, observed-to-expected ratios of 1.07 (95% CI 1.02–1.12) with the extended input and 1.30 (1.24–1.36) with the core input; the calibration slopes were 0.9031 (0.8539–0.9454) and 1.0235 (0.9784–1.0663), the intercepts 0.1587 (0.0433–0.2698) and 0.4860 (0.4011–0.5681), the Brier scores 0.0076 (0.0072–0.0082) and 0.0090 (0.0086–0.0095) and the expected calibration errors 0.0010 (0.0006–0.0017) and 0.0038 (0.0031–0.0045) (Figure S1B, Table S9). In the internal test set the ratios were 0.96 (0.86–1.07) and 0.99 (0.88–1.11) and the slopes 0.8175 (0.7499–0.8997) and 0.9055 (0.8311–0.9899) (Figure S1C, Table S9).

### 3.7. Subgroup and Exploratory Analyses

In the external cohort, discrimination was stable across sex and age strata (extended-input AUROC 0.9541–0.9628, core input 0.9381–0.9531), higher below 65 years; the CSR-transcribed trial estimates showed the same age ordering, whereas the sex ordering was not consistent between input configurations (Table S6). Strata with fewer than ten positive admissions are marked there and not interpreted. Discrimination varied more by ICU than by sex or age: the lowest external AUROC among interpreted strata was 0.9005, with the core input in ICU 1 (Table S6).

Machine learning models trained on the development data—logistic regression, random forest and gradient boosting—ranked significantly below iCARS in every configuration and at every level of analysis (all *p* ≤ 0.001; Table S10).

### 3.8. Variable-Level Contributions

The device presents the 0–100 score and its trajectory only, so variable-level contributions were computed outside it as Shapley additive explanation values on the approved extended-input network. The ordering is dominated by oxygenation, perfusion and consciousness: averaged over the points at which each variable differed from its median fill value, oxygen saturation carried the largest mean absolute contribution, followed by the motor GCS component and systolic blood pressure, then pH and heart rate, with signed contributions broadly consistent with expected physiology (Figure S2). The ordering is specific to pre-arrest points; no-arrest points are reported separately, and no pooled ranking is claimed.

## 4. Discussion

Evaluated unchanged, iCARS discriminated impending in-ICU cardiac arrest with a subject-level AUROC of 0.9428 in a matched confirmatory trial and a prediction-point AUROC of 0.9587 in a fully external cohort spanning 2010–2021, ranking above every conventional comparator and every machine learning baseline trained on the development data. At the prediction-point level, with the extended input, it detected 37.17 and 30.66 percentage points more of the prediction points preceding an arrest than NEWS2 ≥ 7 and SOFA ≥ 11 did (35.86 and 28.10 with the core input), at no greater alarm burden (Table S1).

Validations of machine learning deterioration and cardiac-arrest prediction have largely been retrospective and single-institution [46,49,72], with external or prospective evaluation reported for few systems [55,56,73,74]. The original ward model reported an 82.2% reduction at matched sensitivity against MEWS [54], and the reductions here at matched sensitivity—94% at the NEWS2 anchor and 80% at the SOFA anchor—bracket that figure.

The severity scores differ from the device in what they were built to do: APACHE-II and SAPS-II were developed to adjust mortality risk from the admission window rather than to signal an impending event; APACHE-II has been recalculated serially in published work [75,76], whereas SAPS-II is defined as a single score from the first 24 h [23]. SOFA is scored repeatedly by definition, but its sensitivity at a given cut-off depends on how many organ systems a site can score, so a SOFA cut-off does not transfer unchanged between cohorts. The advantage reported here is also not generic to machine learning, which shows no general benefit over logistic regression in clinical prediction [77].

The core-input configuration uses no laboratory variable and, against the extended input, showed only a modest reduction in discrimination and detected slightly less at matched alarm burden, 77.90% against 80.47% at the SOFA ≥ 11 anchor, at threshold 57 rather than 66 (Table S8); a threshold chosen for one configuration should therefore not be assumed to carry over to the other.

For the clinical deployment of the iCARS model, the trade-off is monotone: more detection requires a lower threshold and a higher alarm burden, while the lead time shortens as the threshold rises (Table S8). Selecting a deployment threshold is therefore a unit-level decision weighed against local prevalence and alarm tolerance, and whether the observed lead time suffices to alter management requires prospective evaluation. A value at or above the unit’s threshold signals a high risk of arrest, a value approaching the threshold warrants closer attention, and the patient’s own trajectory may add context. A unit can set that threshold to match the alarm burden or the sensitivity of a score it already uses, as done here for NEWS2 and SOFA (Tables S1 and S2). Within the 12 h window, iCARS held to the SOFA ≥ 11 alarm budget was above threshold for 74.0% of the observed time before an event, whereas SOFA ≥ 11 was met for 48.7% (70.4% against 41.3% at the NEWS2 ≥ 7 budget; Section 3.3). When the window opened, the extended-input score was already above its matched threshold in 58.3% of event admissions, against 39.7% for SOFA ≥ 11 (53.8% against 31.1% at the NEWS2 ≥ 7 budget), and at threshold 66 the median time from its first threshold crossing in the stay to the event was 80.84 h (Table S14), so the signal often began well before the window in which it was evaluated.

This evaluation has three principal strengths. First, the approved model was evaluated unchanged. Second, the confirmatory trial, although based on previously collected records, used a prespecified success criterion, prespecified endpoints and a sample size determined for that objective within a regulated framework. Third, discrimination remained high in an external cohort from an institution that took no part in the model’s development, over 2010–2021, with alarm burden assessed alongside it.

There are several limitations of this study. First, the evaluation is retrospective: outcomes and predictor values are limited to what the records documented. In the development, internal test, and external data, most events were deaths without CPR (61.1–76.5%), while the trial recorded in-ICU cardiac arrest as a single outcome; do-not-resuscitate status before these deaths was not consistently recorded. Discrimination was lower for arrests with CPR (Section 3.2), and the external event types were classified from structured records without chart review. Second, both institutions are tertiary centres in one country, so transfer to other health systems, hospital types and populations is untested. That said, because the core input needs no laboratory value and still ranked above every comparator in both cohorts (Table 2), it could be used in ICUs with less frequent laboratory testing, such as those of secondary-care hospitals, where its performance remains to be evaluated. Nonetheless, discrimination was maintained in all ICU admissions over twelve years (2010–2021) at an independent institution, which suggests that the model can generalise to other ICU settings. Fairness, likewise, could be examined only in the demographic variables the source datasets carry; within those, discrimination did not differ materially by sex or age (Table S6). Third, the confirmatory trial evaluated one predefined observation window per subject rather than continuous prediction across a whole ICU stay, so its subject-level operating characteristics do not represent alarm burden across a stay; the external cohort addresses that. Case–control sampling can inflate accuracy, most when cases are drawn from the severe end and controls from healthy individuals [68]. In the trial, cases and controls were both drawn at random from all eligible ICU admissions, and each control’s window ended at a random time in the stay, so the control windows were a random sample of the prediction points of admissions without an arrest, which the external cohort includes in full; any such inflation is therefore likely to be small, and the external cohort also showed high discrimination. Fourth, the trial’s case report form does not carry every input the conventional scores require, so several were scored in reduced-item forms, and their trial performance maps only loosely onto the full instruments. Fifth, direct comparison with an ICU-specific real-time cardiac-arrest prediction model was not feasible, because the published system identified for this setting requires inputs these datasets do not contain [46]. Last, whether accuracy of the model changes patient outcomes is a further question that neither cohort here addresses.

This study measures predictive performance and alarm burden, not what deployment does to workflow, treatment decisions, or patient outcomes; prospective implementation evaluation, ideally across more than one health system, remains to be done.

## 5. Conclusions

In a matched confirmatory clinical trial and a large external validation spanning 2010–2021, a fixed, approved machine learning device predicted impending in-ICU cardiac arrest with higher discrimination than the conventional deterioration and severity scores reconstructed here; externally, where most arrests were deaths without CPR, it retained that advantage for arrests with CPR. At the two comparator reference points, NEWS2 ≥ 7 and SOFA ≥ 11, it detected 37.17 and 30.66 percentage points more of the prediction points preceding an arrest at the prediction-point level, with the extended input, at no greater alarm burden, and at no lower sensitivity it generated 16.64-fold and 4.93-fold fewer alarms per bed-day. No operating point is proposed. Prospective implementation studies are needed to determine whether this improves patient outcomes.

## Figures and Tables

**Table 1:** Baseline characteristics of the development, confirmatory-trial and external cohorts.

Characteristic	Development—Arrest Within 12 h (n = 4864)	Development—No Arrest Within 12 h (n = 76,926)	Trial—Arrest Within 12 h (n = 107)	Trial—No Arrest Within 12 h (n = 107)	External—Arrest Within 12 h (n = 2782)	External—No Arrest Within 12 h (n = 27,705)
Cohort and observation						
Patients, n	4723	52,931	107	107	2707	21,656
Prediction points, n	188,585	8353,629	2918	2173	69,079	4,298,696
ICU length of stay, median (IQR), d	3.40 (1.06–10.26)	1.00 (0.59–2.62)	7.55 (3.23–15.48)	14.07 (7.00–24.00)	6.05 (1.86–15.96)	1.35 (0.88–4.45)
Outcome						
Cardiac arrest with CPR, n (%)	1676 (34.46)	0 (0.00)	—	0 (0.00)	653 (23.47)	0 (0.00)
Death without CPR, n (%)	3188 (65.54)	0 (0.00)	—	0 (0.00)	2129 (76.53)	0 (0.00)
Demographics						
Age, mean (SD), years	64.09 (14.98)	61.81 (14.60)	69.31 (14.17)	69.16 (14.79)	64.56 (14.10)	62.56 (14.22)
Aged 65 years or older, n (%)	2696 (55.43)	37,174 (48.32)	75 (70.09)	74 (69.16)	1561 (56.11)	13,458 (48.58)
Male, n (%)	2955 (60.75)	46,667 (60.66)	74 (69.16)	74 (69.16)	1773 (63.73)	16,745 (60.44)
9 vital-sign and GCS variables						
Respiratory rate, /min	24.9 ± 8.2 *	19.7 ± 6.4	21.7 ± 7.0 *	19.2 ± 6.1	18.4 ± 9.7 *	18.8 ± 5.9
Heart rate, /min	93.4 ± 36.9 *	86.9 ± 20.1	92.3 ± 26.6 *	86.0 ± 20.7	89.0 ± 38.4	88.4 ± 19.3
Systolic blood pressure, mmHg	93.8 ± 31.0 *	125.4 ± 24.8	100.1 ± 34.8 *	130.2 ± 29.1	86.6 ± 35.4 *	127.2 ± 24.6
Diastolic blood pressure, mmHg	53.4 ± 18.0 *	70.7 ± 13.8	52.4 ± 16.2 *	64.5 ± 16.4	49.6 ± 18.6 *	67.2 ± 14.6
Body temperature, °C	36.1 ± 1.5 *	36.8 ± 0.7	36.2 ± 0.9 *	36.7 ± 0.7	36.5 ± 0.8 *	36.7 ± 0.4
SpO2, %	84.6 ± 16.6 *	98.1 ± 2.8	93.8 ± 7.7 *	97.7 ± 3.3	87.3 ± 18.0 *	98.6 ± 2.5
GCS–eye	1.83 ± 1.20 *	3.15 ± 1.08	1.72 ± 1.20 *	3.05 ± 1.13	1.88 ± 1.19 *	3.18 ± 0.97
GCS–verbal	3.04 ± 1.62 *	4.47 ± 0.96	2.73 ± 1.46 *	4.39 ± 0.67	2.92 ± 1.54 *	4.18 ± 1.03
GCS–motor	2.60 ± 2.09 *	5.21 ± 1.58	2.54 ± 1.92 *	5.06 ± 1.68	2.69 ± 2.03 *	5.28 ± 1.40
21 laboratory results						
ALT, U/L	389.1 ± 794.1 *	78.3 ± 227.5	536.5 ± 987.8 *	71.2 ± 142.9	330.7 ± 721.7*	73.4 ± 190.3
AST, U/L	781.0 ± 1621.2 *	93.3 ± 342.7	975.6 ± 2015.0 *	105.3 ± 331.7	731.8 ± 1467.8 *	91.7 ± 292.6
Albumin, g/dL	2.56 ± 0.65 *	3.14 ± 0.62	2.71 ± 0.53 *	2.94 ± 0.42	2.58 ± 0.62 *	3.12 ± 0.54
Blood urea nitrogen, mg/dL	37.3 ± 26.6 *	25.4 ± 19.3	37.2 ± 28.3	31.1 ± 19.4	37.5 ± 29.8 *	25.9 ± 20.1
Bilirubin, mg/dL	4.84 ± 7.48 *	1.91 ± 3.59	5.80 ± 7.97 *	2.79 ± 5.59	4.03 ± 6.05 *	2.20 ± 4.41
C-reactive protein, mg/dL	11.5 ± 10.3 *	7.02 ± 8.01	13.1 ± 10.0 *	8.28 ± 8.02	12.0 ± 9.3 *	7.57 ± 7.29
Chloride, mmol/L	101.6 ± 8.8 *	104.5 ± 6.4	99.6 ± 7.4 *	102.0 ± 5.9	103.5 ± 8.2	103.8 ± 5.9
Creatinine, mg/dL	1.94 ± 1.40 *	1.35 ± 1.43	1.70 ± 1.33 *	1.12 ± 0.75	1.85 ± 1.76 *	1.12 ± 1.41
Glucose, mg/dL	169.6 ± 97.9 *	154.3 ± 60.0	149.2 ± 65.7	166.5 ± 89.6	160.5 ± 93.0 *	168.3 ± 71.5
Haemoglobin, g/dL	9.36 ± 2.41 *	10.7 ± 2.1	8.68 ± 1.98 *	9.31 ± 1.81	9.68 ± 2.35 *	10.5 ± 2.0
Prothrombin time, INR	2.16 ± 1.15 *	1.29 ± 0.47	2.11 ± 1.28 *	1.33 ± 0.39	2.37 ± 1.33 *	1.36 ± 0.46
Platelet count, 10^3^/µL	102.4 ± 97.4 *	170.6 ± 103.8	77.8 ± 80.9 *	160.9 ± 135.0	99.1 ± 97.0 *	176.5 ± 117.5
Potassium, mmol/L	4.54 ± 1.16 *	3.98 ± 0.60	4.61 ± 1.14 *	4.01 ± 0.59	4.41 ± 1.15 *	3.84 ± 0.65
Sodium, mmol/L	139.8 ± 8.8 *	137.9 ± 5.7	139.2 ± 7.0 *	136.8 ± 5.3	139.3 ± 8.4 *	136.7 ± 5.7
White cell count, 10^3^/µL	13.9 ± 20.6 *	11.1 ± 8.8	13.1 ± 11.7	12.3 ± 8.4	13.4 ± 19.2 *	10.8 ± 5.5
aPTT, s	63.7 ± 38.9 *	40.7 ± 22.0	54.9 ± 29.6 *	36.2 ± 14.5	72.2 ± 34.5 *	47.0 ± 17.0
Bicarbonate, mmol/L	17.9 ± 6.6 *	25.2 ± 4.8	20.4 ± 8.3 *	27.1 ± 6.3	19.9 ± 6.5 *	25.1 ± 5.3
Lactate, mmol/L	7.92 ± 5.15 *	2.15 ± 2.09	9.24 ± 7.22 *	1.77 ± 1.41	8.74 ± 5.72 *	1.75 ± 1.64
pCO2, mmHg	43.9 ± 19.4 *	39.1 ± 9.9	41.2 ± 19.3	41.4 ± 12.8	43.2 ± 18.1 *	37.4 ± 9.6
pH	7.24 ± 0.16 *	7.42 ± 0.08	7.30 ± 0.13 *	7.44 ± 0.09	7.28 ± 0.15 *	7.44 ± 0.07
pO2, mmHg	103.4 ± 85.4 *	119.6 ± 58.4	96.2 ± 47.0	105.5 ± 37.4	94.8 ± 64.7 *	113.9 ± 46.5

Values are mean ± SD or n (%) unless stated otherwise; vital-sign and laboratory rows are summarised over prediction points (natively observed measurements only). Values outside the plausible ranges of Table S11 are excluded. The arrest columns cover only the prediction points in the 12 h before the event, the other columns the whole observed stay; in the trial, both cover each subject’s single ≤12 h case-report-form window. An admission with no in-window arrest may still carry an event outside the observation window—1557 of 27,705 externally, 1919 of 76,926 in the development cohort, mostly deaths recorded after ICU discharge—and is scored negative throughout. Each patient is counted once in the arrest column if any of their admissions falls in it. A dash indicates that the trial recorded in-ICU cardiac arrest as a single outcome. Further characteristics are in Table S4. An asterisk marks a variable whose two groups differed at *p* < 0.05 within that cohort; cohort, outcome and demographic rows are descriptive and were not tested. ALT = alanine aminotransferase; aPTT = activated partial thromboplastin time; AST = aspartate aminotransferase; INR = international normalised ratio; IQR = interquartile range; pCO2 = partial pressure of carbon dioxide; pO2 = partial pressure of oxygen; SD = standard deviation.

**Table 2:** Discrimination at the 12 h horizon, iCARS versus comparators.

Cohort	Score	n Evaluated	n Positive	AUROC (95% CI)	AUPRC (95% CI)
Trial	iCARS (extended input)	214	107	0.9428 (0.9102–0.9753)	n/a (1:1 matched)
Trial	iCARS (core input)	214	107	0.9103 (0.8716–0.9490)	n/a (1:1 matched)
Trial	mSOFA	214	107	0.7815 (0.7171–0.8390) *	n/a (1:1 matched)
Trial	mAPACHE-II	214	107	0.8583 (0.8012–0.9081) *	n/a (1:1 matched)
Trial	mSAPS-II	214	107	0.8535 (0.7983–0.9032) *	n/a (1:1 matched)
Trial	mNEWS2	214	107	0.8282 (0.7672–0.8788) *	n/a (1:1 matched)
Trial	MEWS	214	107	0.8363 (0.7814–0.8848) *	n/a (1:1 matched)
Trial	CART	214	107	0.7317 (0.6616–0.8008) *	n/a (1:1 matched)
Trial	qSOFA	214	107	0.6836 (0.6166–0.7492) *	n/a (1:1 matched)
External	iCARS (extended input)	4,367,775	69,079	0.9587 (0.9548–0.9626)	0.6922 (0.6597–0.7198)
External	iCARS (core input)	3,754,387	61,073	0.9458 (0.9415–0.9500)	0.6362 (0.6095–0.6608)
External	SOFA	4,367,481	69,068	0.8583 (0.8498–0.8665) *	0.1892 (0.1678–0.2130)
External	APACHE-II	4,367,775	69,079	0.8647 (0.8566–0.8726) *	0.2974 (0.2704–0.3240)
External	SAPS-II	4,367,775	69,079	0.8766 (0.8690–0.8845) *	0.3497 (0.3248–0.3768)
External	NEWS2	4,367,437	69,073	0.8232 (0.8169–0.8295) *	0.1468 (0.1367–0.1577)
External	MEWS	4,367,245	69,064	0.8112 (0.8039–0.8180) *	0.1006 (0.0929–0.1090)
External	CART	4,367,775	69,079	0.7256 (0.7158–0.7357) *	0.0540 (0.0486–0.0599)
External	qSOFA	4,367,195	69,057	0.7161 (0.7071–0.7252) *	0.0411 (0.0376–0.0449)

The prefix m marks a score computed within the 31 case-report-form inputs of the confirmatory trial, a reduced-item set (Section 2.6). The two iCARS rows in the confirmatory trial carry the prespecified Wald intervals transcribed from the clinical study report; computed with the subject-level clustered bootstrap used for every other interval, they are 0.9092–0.9723 (extended input) and 0.8697–0.9458 (core input). Every other interval and *p* value is ours (Section 2.8). An asterisk marks a comparator whose AUROC differs from that of both iCARS configurations in the same cohort at *p* < 0.05, by paired bootstrap on the units scored by both. n evaluated differs between rows because each score has its own missingness. APACHE-II = Acute Physiology and Chronic Health Evaluation II; CART = Cardiac Arrest Risk Triage; CI = confidence interval; iCARS = DeepiCARS; MEWS = Modified Early Warning Score; qSOFA = quick Sequential Organ Failure Assessment; SAPS-II = Simplified Acute Physiology Score II.

## Data Availability

The datasets analysed in this study are not publicly available because of institutional and privacy restrictions but may be made available by the corresponding authors upon reasonable request, subject to approval by the relevant institutions. The analysis code is available from the corresponding authors upon reasonable request. The confirmatory trial protocol and clinical study report are confidential documents of the trial sponsor, VUNO Inc., and are not publicly available; no separate registered protocol was prepared for the internal test set or the external cohort. The model weights and preprocessing of the approved device are proprietary to the manufacturer and are not publicly available.

## Supplementary Materials

The following supporting information can be downloaded at: https://www.mdpi.com/article/10.3390/diagnostics16213671/s1. Figure S1: Calibration of iCARS in the confirmatory trial, the external cohort and the internal test set. Figure S2: Variable-level contributions to the extended-input prediction. Figure S3: Study flow of the internal test set. Table S1: Alarm burden and sensitivity at all four comparator anchors, primary and secondary, the external cohort. Table S2: Threshold sweep and alarm burden, the external cohort. Table S3: Sensitivity at a fixed alarm volume, the external cohort. Table S4: ICU category, total observation and severity of illness. Table S5: Discrimination by prediction horizon and input configuration. Table S6: Subgroup AUROC at the 12 h horizon. Table S7: SAPS-II scored as originally defined against the repeated form. Table S8: iCARS operating characteristics at the anchor-matched thresholds, the external cohort. Table S9: Calibration in the confirmatory trial, the internal test set and the external cohort. Table S10: Machine learning benchmark models trained on the development set, fitted without hyperparameter tuning. Table S11: Plausible ranges applied to the reported input distributions. Table S12: Alarm burden under the two row-set conventions, the external cohort. Table S13: Discrimination with the positive set restricted by event type, the internal test set and the external cohort. Table S14: Discrimination, operating characteristics and timing with the pre-window points of event admissions counted as negatives, the external cohort. Table S15: Discrimination and sensitivity by interval before the event. Table S16: External event records and discrimination for arrests with CPR before and after the 2018 legislation on life-sustaining treatment decisions. File S1: Completed TRIPOD+AI checklist. File S2: completed TRIPOD+AI for Abstracts checklist.
