# Supplementary material for: Real-Time Machine Learning Model to Predict Cardiac Arrest Within 12 Hours in Critically Ill Patients (DeepiCARS): A Confirmatory Trial and External Validation
Source: Diagnostics (Basel). 2026 Nov 6;16(21):3671. doi: 10.3390/diagnostics16213671 (PMC13762413; doi:10.3390/diagnostics16213671)
Supplement: Supplementary file 1 [file diagnostics-16-03671-s001.zip › File_S2_TRIPOD_AI_abstracts_checklist.pdf]

Version: 11-January-2024

Item	Section	Checklist item	Reported in	Status
**TITLE**				
1	Title	Identify the study as developing or evaluating the performance of a multivariable prediction model, the target population, and the outcome to be predicted	Title	Yes
**BACKGROUND**				
2	Background	Provide a brief explanation of the healthcare context and rationale for developing or evaluating the performance of all models	Abstract	Yes
3	Objectives	Specify the study objectives, including whether the study describes model development, evaluation, or both	Abstract	Yes
**METHODS**				
4	Methods	Describe the sources of data	Abstract	Yes
5	Methods	Describe the eligibility criteria and setting where the data were collected	Abstract	Yes
6	Methods	Specify the outcome to be predicted, including time horizon of predictions in case of prognostic models	Abstract	Yes
7	Methods	Specify the type of model, a summary of the model-building steps, and the method for internal validation†	Abstract	Yes
8	Methods	Specify the measures used to assess model performance (e.g., discrimination, calibration, clinical utility)	Abstract	Yes
**RESULTS**				
9	Results	Report the number of participants and outcome events	Abstract	Yes
10	Results	Summarise the predictors in the final model†	Abstract	Yes
11	Results	Report model performance estimates (with confidence intervals)	Abstract	Yes
**DISCUSSION**				
12	Discussion	Give an overall interpretation of the main results	Abstract	Yes
**OTHER**				
13	Registration	Give the registration number and name of the registry or repository	Abstract	Yes

From: Collins GS, Moons KGM, Dhiman P, et al. BMJ 2024;385:e078378. doi:10.1136/bmj-2023-078378 (TRIPOD+AI for Abstracts, Table 3)

† Relevant only to studies describing the development of a prediction model; this study describes the model's development and its evaluation, so items 7 and 10 are reported.

Locations are given as the manuscript section rather than a page number, so that the entry remains valid across typesetting. Abstract means the structured abstract of the manuscript.

Status key: **Yes** = reported in the abstract. The abstract states, for item 13, that the trial, assigning no intervention, required no registration, which the item accepts in place of a registration number.
