# Supplementary material for: Real-Time Machine Learning Model to Predict Cardiac Arrest Within 12 Hours in Critically Ill Patients (DeepiCARS): A Confirmatory Trial and External Validation
Source: Diagnostics (Basel). 2026 Nov 6;16(21):3671. doi: 10.3390/diagnostics16213671 (PMC13762413; doi:10.3390/diagnostics16213671)
Supplement: Supplementary file 1 [file diagnostics-16-03671-s001.zip › Supplementary_Materials_iCARS.pdf]

Supplementary Materials

Real-time machine learning model to predict cardiac arrest within 12 hours in critically ill patients (DeepiCARS): a confirmatory trial and external validation

Ukdong Gim, Leerang Lim, Kyung-Jae Cho, Woo Hyun Cho and Hyung-Chul Lee

Reference numbers in square brackets and section numbers refer to the main article. iCARS denotes DeepiCARS throughout.

Contents

Figure S1. Calibration of iCARS in the confirmatory trial, the external cohort and the internal test set.

Figure S2. Variable-level contributions to the extended-input prediction.

Figure S3. Study flow of the internal test set, drawn on the conventions of Figure 1.

Table S1. Alarm burden and sensitivity at all four comparator anchors, primary and secondary, the external cohort.

Table S2. Threshold sweep and alarm burden, the external cohort.

Table S3. Sensitivity at a fixed alarm volume, the external cohort.

Table S4. ICU category, total observation and severity of illness.

Table S5. Discrimination by prediction horizon and input configuration.

Table S6. Subgroup AUROC at the 12-hour horizon.

Table S7. SAPS-II scored as originally defined against the repeated form.

Table S8. iCARS operating characteristics at the anchor-matched thresholds, the external cohort.

Table S9. Calibration in the confirmatory trial, the internal test set and the external cohort.

Table S10. Machine-learning benchmark models trained on the development set, fitted without hyperparameter tuning.

Table S11. Plausible ranges applied to the reported input distributions.

Table S12. Alarm burden under the two row-set conventions, the external cohort.

Table S13. Discrimination with the positive set restricted by event type, the internal test set and the external cohort.

Table S14. Discrimination, operating characteristics and timing with the pre-window points of event admissions counted as negatives, the external cohort.

Table S15. Discrimination and sensitivity by interval before the event.

Table S16. External event records and discrimination for arrests with CPR before and after the 2018 legislation on life-sustaining treatment decisions.

Supplementary Figures

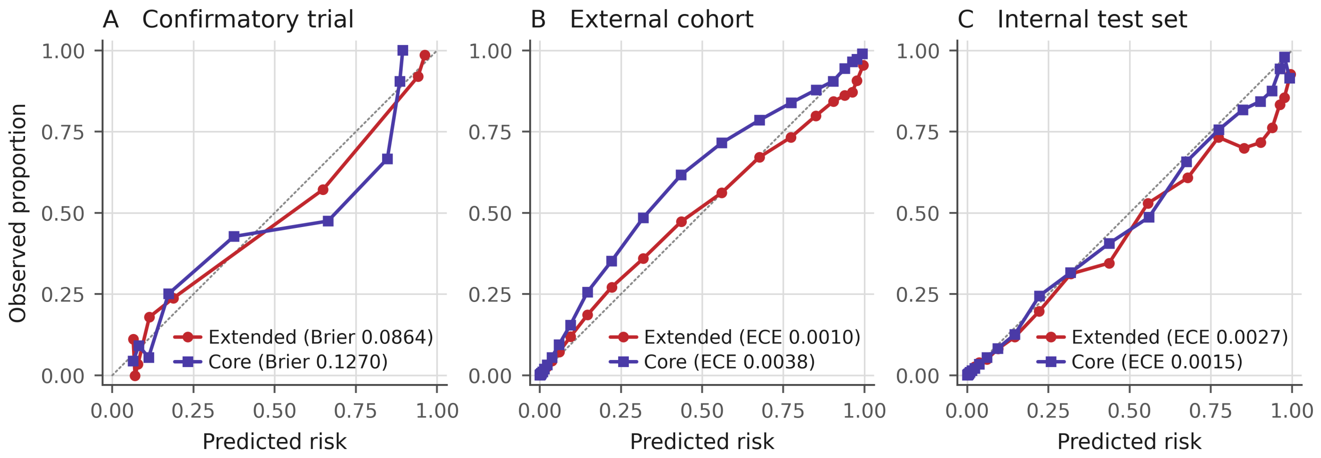

Figure S1. Calibration of iCARS in the confirmatory trial, the external cohort and the internal test set. The dotted diagonal is perfect calibration, and each panel overlays the extended and core inputs. Panel A is taken from the calibration plot in the clinical study report: the points are the report's own binned subject-level estimates (214 subjects) on the device's output, and the trial's 1:1 design fixes the observed event fraction at 50%. Panels B and C show the absolute 12-hour risk of Section 2.3, with its calibration estimated on the development data and applied unchanged, at the prediction-point level in the external cohort and the internal test set; each point is a bin of predicted risk, equally wide on the logit scale, and bins with fewer than 200 points are omitted. The legends give the Brier scores printed in the report (panel A) and the expected calibration errors of Table S9 (panels B and C); statistics with confidence intervals are in Table S9. ECE, expected calibration error.

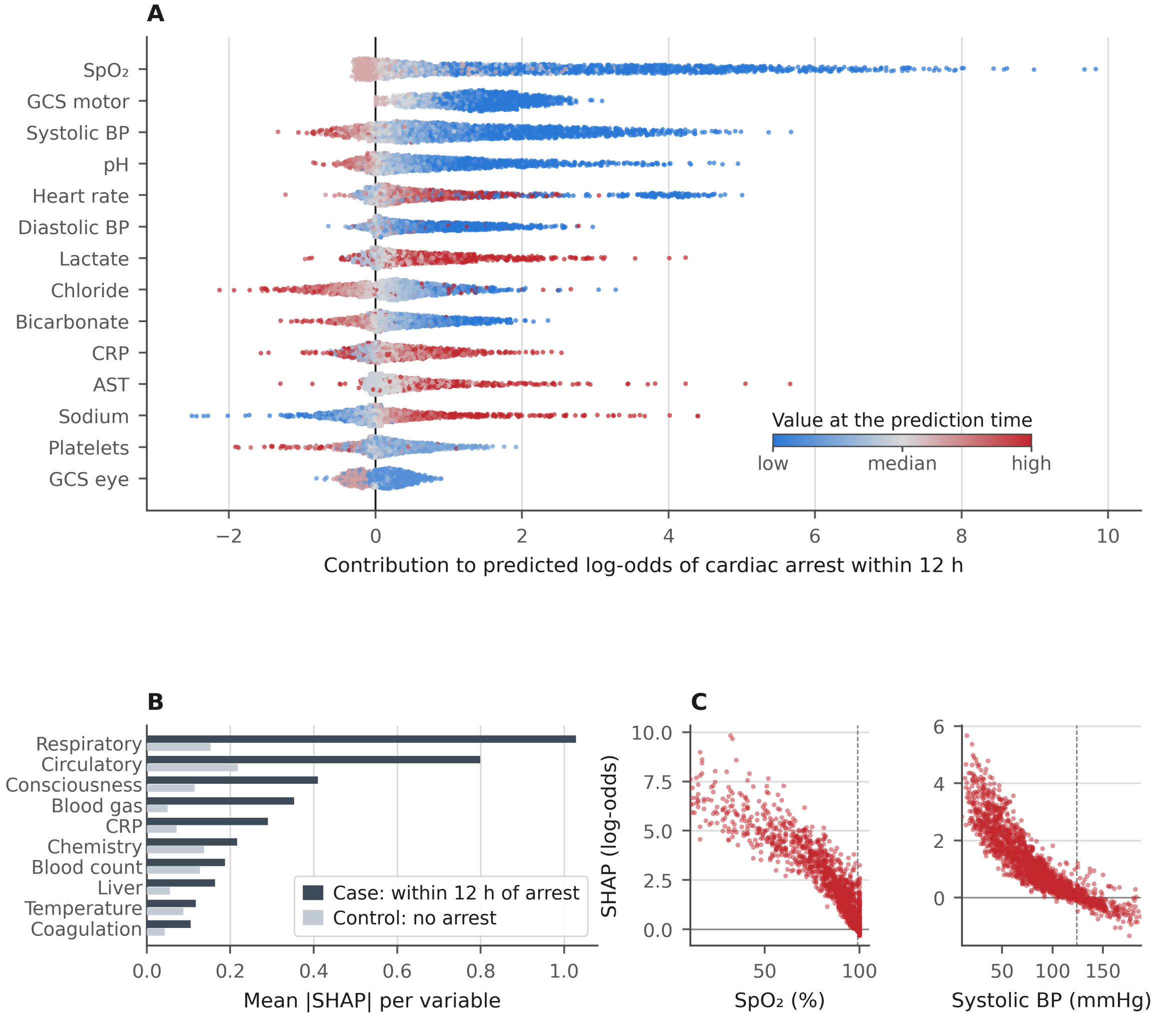

Figure S2. Variable-level contributions to the extended-input prediction. Shapley additive explanation values, computed post hoc in the external cohort on 5,000 prediction points — 2,000 within 12 hours of an arrest, 3,000 from admissions with no arrest. Panel A, the fourteen variables with the largest mean absolute contribution over the pre-arrest points at which each differed from its median fill value; one mark is one such point. Panel B, contributions at every point, averaged over the variables of each clinical block, for pre-arrest and no-arrest points separately. Panel C, contribution against recorded value for oxygen saturation and systolic blood pressure; the dashed line is the median held in the device's own preprocessing statistics, supplied when an input has not yet been observed.

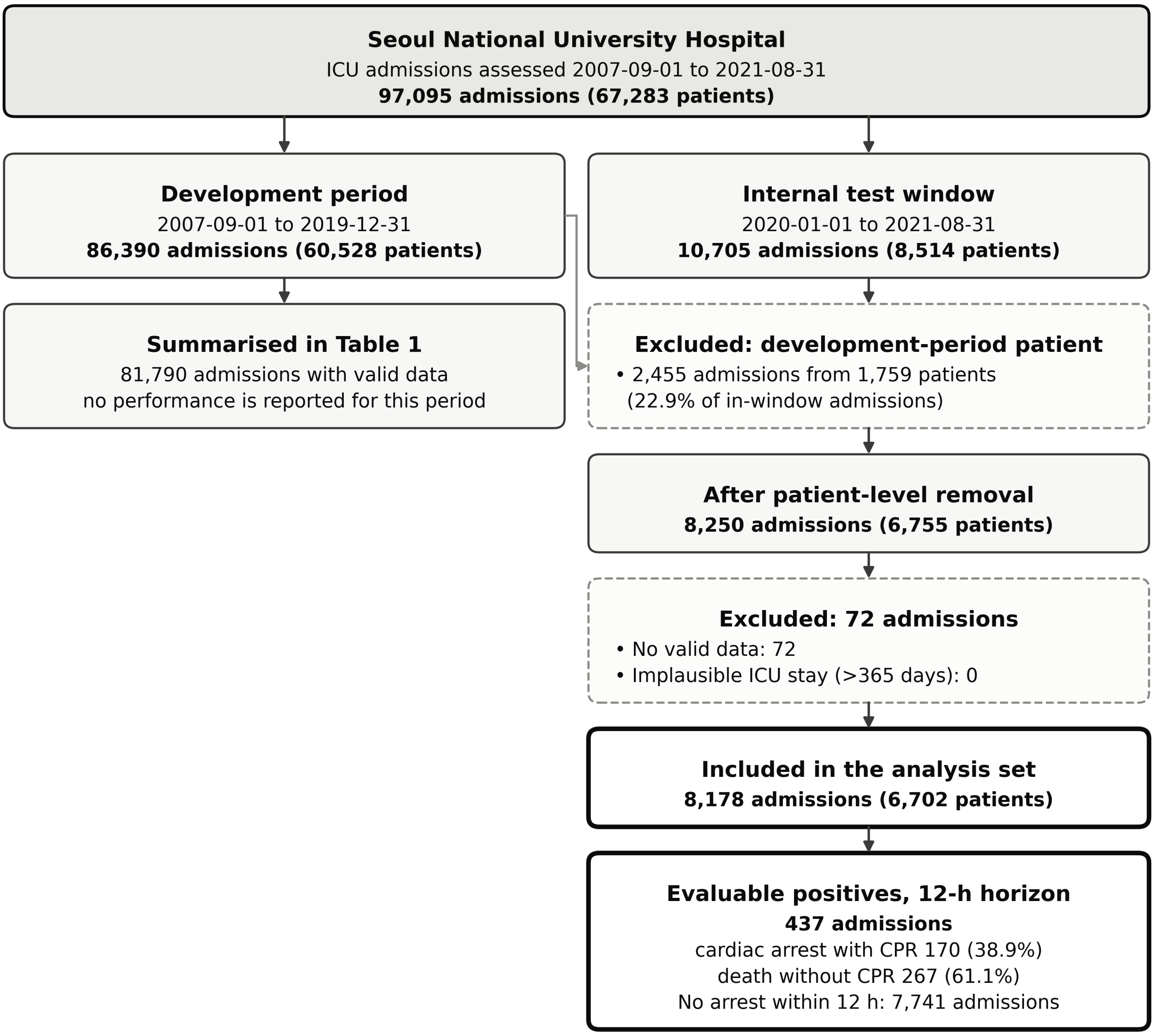

Figure S3. Study flow of the internal test set, drawn on the conventions of Figure 1. Patient counts do not add across the two branches (60,528 + 8,514 − 1,759 = 67,283) because 1,759 patients were admitted in both periods. The data-integrity exclusions are grouped as in Figure 1. The 166 event admissions with no evaluable positive point, mostly deaths more than 12 hours after ICU discharge, are scored negative throughout, as in Table 1.

Supplementary Tables

Table S1. Alarm burden and sensitivity at all four comparator anchors, primary and secondary, the external cohort.

(a) Extended input.

Comparator anchor	Readout	Comparator sensitivity (%)	Comparator MACPD	Matched iCARS threshold	iCARS sensitivity (%)	iCARS MACPD	Sensitivity difference, pp	MACPD ratio, fold
NEWS2 ≥7 (primary)	equal alarm rate (primary)	40.19 (38.99–41.32)	0.7570 (0.7401–0.7734)	76	77.36 (76.04–78.59)	0.2666 (0.2552–0.2783)	37.17 (35.75–38.65)	2.84× (2.73–2.95)
NEWS2 ≥7 (primary)	equal sensitivity (secondary)	40.19 (38.99–41.32)	0.7570 (0.7401–0.7734)	100	41.62 (40.07–43.21)	0.0455 (0.0424–0.0488)	1.43 (-0.31 to 3.35)	16.64× (15.53–17.79)
SOFA ≥11 (primary)	equal alarm rate (primary)	49.81 (47.91–51.72)	0.3411 (0.3239–0.3584)	66	80.47 (79.19–81.60)	0.3372 (0.3243–0.3503)	30.66 (28.76–32.65)	1.01× (0.96–1.06)
SOFA ≥11 (primary)	equal sensitivity (secondary)	49.81 (47.91–51.72)	0.3411 (0.3239–0.3584)	99	52.29 (50.67–53.81)	0.0693 (0.0651–0.0736)	2.48 (0.37–4.58)	4.93× (4.60–5.28)
NEWS2 ≥6 (secondary)	equal alarm rate (primary)	57.35 (56.11–58.52)	1.2063 (1.1857–1.2263)	48	84.85 (83.74–85.86)	0.4852 (0.4694–0.5015)	27.51 (26.37–28.76)	2.49× (2.41–2.56)
NEWS2 ≥6 (secondary)	equal sensitivity (secondary)	57.35 (56.11–58.52)	1.2063 (1.1857–1.2263)	97	59.89 (58.34–61.44)	0.0992 (0.0936–0.1051)	2.55 (1.05–4.14)	12.16× (11.50–12.85)
SOFA ≥10 (secondary)	equal alarm rate (primary)	57.39 (55.56–59.20)	0.4500 (0.4298–0.4698)	52	83.97 (82.81–85.03)	0.4481 (0.4329–0.4638)	26.58 (24.80–28.49)	1.00× (0.96–1.05)
SOFA ≥10 (secondary)	equal sensitivity (secondary)	57.39 (55.56–59.20)	0.4500 (0.4298–0.4698)	97	59.89 (58.34–61.44)	0.0992 (0.0936–0.1051)	2.51 (0.53–4.50)	4.54× (4.28–4.82)

(b) Core input.

Comparator anchor	Readout	Comparator sensitivity (%)	Comparator MACPD	Matched iCARS threshold	iCARS sensitivity (%)	iCARS MACPD	Sensitivity difference, pp	MACPD ratio, fold
NEWS2 ≥7 (primary)	equal alarm rate (primary)	40.19 (38.99–41.32)	0.7570 (0.7401–0.7734)	62	76.04 (74.71–77.27)	0.2962 (0.2851–0.3079)	35.86 (34.44–37.38)	2.56× (2.48–2.64)
NEWS2 ≥7 (primary)	equal sensitivity (secondary)	40.19 (38.99–41.32)	0.7570 (0.7401–0.7734)	98	42.10 (40.48–43.73)	0.0433 (0.0407–0.0460)	1.91 (0.08–3.76)	17.48× (16.54–18.52)
SOFA ≥11 (primary)	equal alarm rate (primary)	49.81 (47.91–51.72)	0.3411 (0.3239–0.3584)	57	77.90 (76.63–79.08)	0.3401 (0.3281–0.3530)	28.10 (26.06–30.17)	1.00× (0.95–1.06)
SOFA ≥11 (primary)	equal sensitivity (secondary)	49.81 (47.91–51.72)	0.3411 (0.3239–0.3584)	95	50.90 (49.27–52.49)	0.0660 (0.0622–0.0700)	1.10 (-1.03 to 3.45)	5.16× (4.84–5.50)
NEWS2 ≥6 (secondary)	equal alarm rate (primary)	57.35 (56.11–58.52)	1.2063 (1.1857–1.2263)	40	83.85 (82.71–84.85)	0.5225 (0.5073–0.5393)	26.50 (25.34–27.67)	2.31× (2.25–2.37)
NEWS2 ≥6 (secondary)	equal sensitivity (secondary)	57.35 (56.11–58.52)	1.2063 (1.1857–1.2263)	90	58.36 (56.76–59.94)	0.0993 (0.0941–0.1049)	1.02 (-0.39 to 2.52)	12.14× (11.54–12.78)

Comparator anchor	Readout	Comparator sensitivity (%)	Comparator MACPD	Matched iCARS threshold	iCARS sensitivity (%)	iCARS MACPD	Sensitivity difference, pp	MACPD ratio, fold
SOFA ≥10 (secondary)	equal alarm rate (primary)	57.39 (55.56–59.20)	0.4500 (0.4298–0.4698)	46	81.79 (80.57–82.86)	0.4497 (0.4357–0.4653)	24.40 (22.48–26.38)	1.00× (0.96–1.05)
SOFA ≥10 (secondary)	equal sensitivity (secondary)	57.39 (55.56–59.20)	0.4500 (0.4298–0.4698)	90	58.36 (56.76–59.94)	0.0993 (0.0941–0.1049)	0.98 (-1.15 to 3.23)	4.53× (4.28–4.79)

iCARS thresholds are on the scale of Section 2.3. Each anchor is read twice (Section 2.7): at equal alarm rate, the matched threshold is the lowest whose burden is at or below the comparator's under both the 8-hour-block and the raw definition; at equal sensitivity, the highest reaching the comparator's sensitivity, where the MACPD ratio is a lower bound. In panel (b) the two sensitivities rest on different prediction-point sets, because the core input cannot be scored at every point. MACPD is above-threshold 8-hour blocks per bed-day; it and sensitivity rest on different row sets (Table S12). Parenthetical ranges are 95% percentile confidence intervals from patient-level clustered bootstrap with 2,000 resamples, each ratio and difference formed within the resample and conditional on the matched threshold. Matched instead on the continuous probability output, which makes the equal-sensitivity match exact and does not depend on the calibration of Section 2.3, the MACPD ratios at NEWS2 ≥7, SOFA ≥11, NEWS2 ≥6 and SOFA ≥10 are 17.61× (16.44–18.84), 5.48× (5.10–5.88), 13.86× (13.08–14.68) and 5.16× (4.85–5.50) with the extended input and 19.16× (18.12–20.29), 5.45× (5.11–5.81), 12.87× (12.22–13.56) and 4.79× (4.52–5.07) with the core input.

Table S2. Threshold sweep and alarm burden, the external cohort.

Series	Cut-off	Role	Sensitivity (%)	Specificity (%)	PPV (%)	NNE	MACPD (8-h blocks/bed-day)	MACPD (raw)
iCARS, extended	48	NEWS2 ≥6, equal alarm rate	84.85 (83.74–85.86)	95.04 (94.75–95.31)	21.58 (20.25–22.94)	4.63 (4.36–4.94)	0.485 (0.469–0.501)	4.243 (4.064–4.432)
iCARS, extended	50		84.41 (83.28–85.45)	95.36 (95.07–95.61)	22.63 (21.24–24.05)	4.42 (4.16–4.71)	0.466 (0.450–0.482)	4.074 (3.897–4.258)
iCARS, extended	52	SOFA ≥10, equal alarm rate	83.97 (82.81–85.03)	95.66 (95.38–95.90)	23.71 (22.27–25.20)	4.22 (3.97–4.49)	0.448 (0.433–0.464)	3.912 (3.741–4.094)
iCARS, extended	60		82.10 (80.87–83.18)	96.70 (96.46–96.91)	28.54 (26.77–30.33)	3.50 (3.30–3.74)	0.383 (0.369–0.397)	3.325 (3.171–3.490)
iCARS, extended	66	SOFA ≥11, equal alarm rate	80.47 (79.19–81.60)	97.34 (97.13–97.53)	32.74 (30.69–34.78)	3.05 (2.87–3.26)	0.337 (0.324–0.350)	2.930 (2.787–3.083)
iCARS, extended	70		79.35 (78.07–80.52)	97.73 (97.54–97.90)	35.95 (33.71–38.13)	2.78 (2.62–2.97)	0.309 (0.296–0.321)	2.680 (2.543–2.826)
iCARS, extended	76	NEWS2 ≥7, equal alarm rate	77.36 (76.04–78.59)	98.24 (98.08–98.38)	41.44 (38.87–43.89)	2.41 (2.28–2.57)	0.267 (0.255–0.278)	2.318 (2.194–2.452)
iCARS, extended	80		75.85 (74.47–77.10)	98.55 (98.40–98.68)	45.74 (42.96–48.37)	2.19 (2.07–2.33)	0.239 (0.228–0.249)	2.082 (1.967–2.203)
iCARS, extended	90		69.86 (68.37–71.23)	99.24 (99.13–99.32)	59.50 (56.03–62.73)	1.68 (1.59–1.78)	0.166 (0.158–0.174)	1.469 (1.381–1.560)
iCARS, extended	95		64.03 (62.53–65.52)	99.55 (99.47–99.62)	69.74 (65.93–73.21)	1.43 (1.37–1.52)	0.121 (0.115–0.128)	1.086 (1.016–1.162)
iCARS, extended	96		62.21 (60.68–63.72)	99.62 (99.54–99.68)	72.34 (68.44–75.89)	1.38 (1.32–1.46)	0.111 (0.105–0.117)	0.992 (0.926–1.064)
iCARS, extended	97	NEWS2 ≥6, equal sensitivity; SOFA ≥10, equal sensitivity	59.89 (58.34–61.44)	99.69 (99.62–99.74)	75.43 (71.36–78.96)	1.33 (1.27–1.40)	0.099 (0.094–0.105)	0.888 (0.826–0.955)
iCARS, extended	98		56.94 (55.37–58.52)	99.76 (99.69–99.80)	78.94 (74.79–82.48)	1.27 (1.21–1.34)	0.085 (0.080–0.091)	0.768 (0.712–0.828)
iCARS, extended	99	SOFA ≥11, equal sensitivity	52.29 (50.67–53.81)	99.83 (99.78–99.86)	82.95 (78.78–86.35)	1.21 (1.16–1.27)	0.069 (0.065–0.074)	0.623 (0.577–0.675)
iCARS, extended	100	NEWS2 ≥7, equal sensitivity	41.62 (40.07–43.21)	99.92 (99.88–99.95)	89.33 (85.00–92.59)	1.12 (1.08–1.18)	0.045 (0.042–0.049)	0.399 (0.366–0.438)
iCARS, core	40	NEWS2 ≥6, equal alarm rate	83.85 (82.71–84.85)	92.56 (92.17–92.92)	15.70 (14.73–16.76)	6.37 (5.97–6.79)	0.522 (0.507–0.539)	4.187 (4.022–4.365)
iCARS, core	46	SOFA ≥10, equal alarm rate	81.79 (80.57–82.86)	94.08 (93.74–94.41)	18.61 (17.43–19.88)	5.37 (5.03–5.74)	0.450 (0.436–0.465)	3.566 (3.415–3.727)

Series	Cut-off	Role	Sensitivity (%)	Specificity (%)	PPV (%)	NNE	MACPD (8-h blocks/bed-day)	MACPD (raw)
iCARS, core	50		80.46 (79.21–81.58)	94.94 (94.63–95.23)	20.81 (19.50–22.25)	4.80 (4.49–5.13)	0.406 (0.393–0.421)	3.206 (3.067–3.359)
iCARS, core	57	SOFA ≥11, equal alarm rate	77.90 (76.63–79.08)	96.17 (95.91–96.41)	25.15 (23.55–26.92)	3.98 (3.71–4.25)	0.340 (0.328–0.353)	2.657 (2.532–2.793)
iCARS, core	60		76.78 (75.48–78.01)	96.62 (96.38–96.85)	27.29 (25.55–29.20)	3.66 (3.42–3.91)	0.313 (0.302–0.326)	2.447 (2.328–2.575)
iCARS, core	62	NEWS2 ≥7, equal alarm rate	76.04 (74.71–77.27)	96.90 (96.67–97.12)	28.85 (27.00–30.88)	3.47 (3.24–3.70)	0.296 (0.285–0.308)	2.312 (2.197–2.434)
iCARS, core	70		72.47 (71.05–73.77)	97.85 (97.67–98.03)	35.82 (33.52–38.20)	2.79 (2.62–2.98)	0.234 (0.224–0.244)	1.825 (1.728–1.930)
iCARS, core	80		66.93 (65.49–68.35)	98.78 (98.65–98.91)	47.63 (44.58–50.64)	2.10 (1.97–2.24)	0.164 (0.156–0.171)	1.291 (1.215–1.372)
iCARS, core	90	NEWS2 ≥6, equal sensitivity; SOFA ≥10, equal sensitivity	58.36 (56.76–59.94)	99.47 (99.37–99.55)	64.47 (60.36–68.36)	1.55 (1.46–1.66)	0.099 (0.094–0.105)	0.802 (0.748–0.857)
iCARS, core	95	SOFA ≥11, equal sensitivity	50.90 (49.27–52.49)	99.74 (99.67–99.79)	76.25 (71.76–80.33)	1.31 (1.24–1.39)	0.066 (0.062–0.070)	0.548 (0.509–0.587)
iCARS, core	96		48.67 (47.08–50.30)	99.79 (99.73–99.84)	79.21 (74.68–83.29)	1.26 (1.20–1.34)	0.059 (0.056–0.063)	0.490 (0.455–0.526)
iCARS, core	97		45.89 (44.28–47.52)	99.84 (99.78–99.88)	82.33 (77.81–86.30)	1.21 (1.16–1.29)	0.052 (0.049–0.055)	0.428 (0.396–0.460)
iCARS, core	98	NEWS2 ≥7, equal sensitivity	42.10 (40.48–43.73)	99.88 (99.84–99.92)	85.55 (81.00–89.36)	1.17 (1.12–1.23)	0.043 (0.041–0.046)	0.359 (0.333–0.386)
iCARS, core	99		36.99 (35.44–38.60)	99.93 (99.89–99.95)	89.22 (84.86–92.76)	1.12 (1.08–1.18)	0.034 (0.032–0.036)	0.277 (0.256–0.299)
iCARS, core	100		26.75 (25.25–28.32)	99.97 (99.95–99.98)	94.02 (90.60–96.65)	1.06 (1.03–1.10)	0.021 (0.020–0.022)	0.161 (0.148–0.176)
NEWS2 ≥7	7	primary anchor	40.19 (38.99–41.32)	95.65 (95.47–95.81)	12.92 (12.14–13.73)	7.74 (7.28–8.24)	0.757 (0.740–0.773)	2.321 (2.248–2.404)
SOFA ≥11	11	primary anchor	49.81 (47.91–51.72)	95.18 (94.77–95.56)	14.24 (13.02–15.58)	7.02 (6.42–7.68)	0.341 (0.324–0.358)	3.478 (3.289–3.675)
NEWS2 ≥6	6	secondary anchor	57.35 (56.11–58.52)	91.19 (90.90–91.45)	9.47 (8.91–10.02)	10.56 (9.98–11.22)	1.206 (1.186–1.226)	4.271 (4.157–4.393)
SOFA ≥10	10	secondary anchor	57.39 (55.56–59.20)	92.79 (92.27–93.27)	11.34 (10.46–12.29)	8.82 (8.14–9.56)	0.450 (0.430–0.470)	4.679 (4.454–4.917)

The iCARS sweep is tabulated from 50 upward, with any matched threshold below 50 added: below 50 specificity falls away and the alarm burden climbs steeply. MACPD (raw) counts individual above-threshold prediction points per bed-day rather than blocks. PPV, the number needed to evaluate and MACPD rest on different row sets and should not be combined (Table S12). Parenthetical ranges are 95% confidence intervals as in Table S1.

Table S3. Sensitivity at a fixed alarm volume, the external cohort.

(a) Extended input. Budget 0.3372 above-threshold 8-hour blocks per bed-day, at iCARS threshold 66; iCARS sensitivity 80.47%.

Comparator	Most sensitive cut-off within the budget	Comparator MACPD	Comparator sensitivity (%)	Sensitivity ratio, fold (iCARS/comparator)
SOFA (full, upper-bound cardiovascular)	12	0.255 (0.240–0.270)	42.24 (40.36–44.18)	1.90× (1.83–1.99)
NEWS2 (7-item)	9	0.226 (0.219–0.234)	21.17 (20.26–22.06)	3.80× (3.64–3.97)
MEWS	7	0.174 (0.167–0.181)	13.98 (13.24–14.67)	5.75× (5.49–6.08)
APACHE-II (full)	20	0.307 (0.292–0.323)	51.19 (49.46–52.89)	1.57× (1.52–1.62)
SAPS-II (full)	47	0.322 (0.306–0.338)	55.14 (53.39–56.85)	1.46× (1.42–1.50)
CART	31	0.304 (0.289–0.319)	11.88 (11.04–12.75)	6.77× (6.32–7.30)
qSOFA	3	0.191 (0.182–0.201)	10.83 (9.93–11.76)	7.43× (6.86–8.12)

(b) Core input. Budget 0.3401 above-threshold 8-hour blocks per bed-day, at iCARS threshold 57; iCARS sensitivity 77.90%.

Comparator	Most sensitive cut-off within the budget	Comparator MACPD	Comparator sensitivity (%)	Sensitivity ratio, fold (iCARS/comparator)
SOFA (full, upper-bound cardiovascular)	12	0.255 (0.240–0.270)	42.24 (40.36–44.18)	1.84× (1.76–1.93)
NEWS2 (7-item)	9	0.226 (0.219–0.234)	21.17 (20.26–22.06)	3.68× (3.53–3.83)
MEWS	7	0.174 (0.167–0.181)	13.98 (13.24–14.67)	5.57× (5.31–5.88)
APACHE-II (full)	20	0.307 (0.292–0.323)	51.19 (49.46–52.89)	1.52× (1.47–1.57)
SAPS-II (full)	47	0.322 (0.306–0.338)	55.14 (53.39–56.85)	1.41× (1.37–1.46)
CART	31	0.304 (0.289–0.319)	11.88 (11.04–12.75)	6.56× (6.12–7.05)
qSOFA	3	0.191 (0.182–0.201)	10.83 (9.93–11.76)	7.19× (6.62–7.87)

Table S1’s equal-alarm-rate reading with the roles reversed (Section 2.7): the iCARS burden at the threshold matched to SOFA ≥11 (Table S8) is the budget, and each comparator is given its most sensitive cut-off whose burden does not exceed it. The slack runs the other way here — the comparator falls short of the budget rather than meeting it — so this ratio overstates rather than understates the difference at strictly equal alarm volume. Parenthetical ranges are 95% confidence intervals as in Table S1, conditional on the cut-off shown.

Table S4. ICU category, total observation and severity of illness.

Characteristic	Development — arrest within 12 h (n=4,864)	Development — no arrest within 12 h (n=76,926)	Trial — arrest within 12 h (n=107)	Trial — no arrest within 12 h (n=107)	External — arrest within 12 h (n=2,782)	External — no arrest within 12 h (n=27,705)
ICU group and total observation						
ICU group [MICU], n (%)	2,316 (47.62)	5,331 (6.93)	39 (36.45)	39 (36.45)	3 (0.11)	23 (0.08)
ICU group [SICU family], n (%)	901 (18.52)	31,474 (40.91)	25 (23.36)	25 (23.36)	9 (0.32)	414 (1.49)
ICU group [EICU], n (%)	1,306 (26.85)	7,933 (10.31)	43 (40.19)	43 (40.19)	763 (27.43)	6,046 (21.82)
ICU group [other], n (%)	341 (7.01)	32,188 (41.84)	0 (0.00)	0 (0.00)	2,007 (72.14)	21,222 (76.60)
Total observation, bed-days	32,205.5	194,064.7	51.5	52.7	27,937.8	123,080.5
Severity in the first 24 h						
APACHE-II, mean (SD)	-	-	-	-	18.91 (6.95)	11.71 (4.72)
SAPS-II, mean (SD)	-	-	-	-	44.26 (14.31)	25.55 (11.03)
SOFA, mean (SD)	-	-	-	-	8.80 (3.98)	5.26 (2.97)

Baseline characteristics beyond those in Table 1. At the development site *SICU family* groups SICU with SICU2 (and CPICU in the trial) and *other* collects the coronary and respiratory units; the constituent units differ at the external institution. Severity scores are reconstructed from electronic health record data, not hospital-recorded values. Only the external cohort's records carry every item the full instruments require, so full-item scores are external only (hyphen elsewhere); the reduced forms reconstructed for the confirmatory trial, marked with the prefix m, are in Table 2 and supported 5 of 6 SOFA organ systems, 13 of 14 APACHE-II components, 11 of 17 SAPS-II components and 6 of 7 NEWS2 parameters, MEWS, CART and qSOFA being the full instruments in both. Columns are formed as in Table 1.

Table S5. Discrimination by prediction horizon and input configuration.

Cohort	Input	Horizon (h)	Unit	n	n positive	AUROC (95% CI)	AUPRC (95% CI)	Note
Confirmatory trial	extended	3	subject	214	107	0.9383 (0.9052, 0.9715) [CSR]	-	
Confirmatory trial	extended	6	subject	214	107	0.9356 (0.9015, 0.9697) [CSR]	-	
Confirmatory trial	extended	9	subject	214	107	0.9312 (0.8954, 0.9670) [CSR]	-	co-primary endpoint [CSR]
Confirmatory trial	extended	12	subject	214	107	0.9428 (0.9102, 0.9753) [CSR]	-	
Confirmatory trial	core	3	subject	214	107	0.9320 (0.8994, 0.9646) [CSR]	-	
Confirmatory trial	core	6	subject	214	107	0.9255 (0.8902, 0.9608) [CSR]	-	
Confirmatory trial	core	9	subject	214	107	0.9233 (0.8883, 0.9583) [CSR]	-	
Confirmatory trial	core	12	subject	214	107	0.9103 (0.8716, 0.9490) [CSR]	-	co-primary endpoint [CSR]
External cohort	extended	3	prediction point	4,324,378	25,682	0.9744 (0.9712–0.9774)	0.7271 (0.6799–0.7641)	
External cohort	extended	6	prediction point	4,339,390	40,694	0.9669 (0.9634–0.9704)	0.7060 (0.6674–0.7392)	
External cohort	extended	9	prediction point	4,353,818	55,122	0.9622 (0.9584–0.9660)	0.6984 (0.6637–0.7268)	
External cohort	extended	12	prediction point	4,367,775	69,079	0.9587 (0.9548–0.9626)	0.6922 (0.6597–0.7198)	
External cohort	core	3	prediction point	3,717,403	24,089	0.9709 (0.9678–0.9739)	0.7203 (0.6896–0.7495)	
External cohort	core	6	prediction point	3,730,427	37,113	0.9595 (0.9556–0.9631)	0.6761 (0.6475–0.7035)	
External cohort	core	9	prediction point	3,742,712	49,398	0.9521 (0.9478–0.9561)	0.6536 (0.6238–0.6802)	
External cohort	core	12	prediction point	3,754,387	61,073	0.9458 (0.9415–0.9500)	0.6362 (0.6095–0.6608)	

Levels of analysis differ by cohort — the confirmatory trial per subject, the external cohort per prediction point (Unit column) — and the two cohorts are not compared with each other. Values tagged [CSR], including their confidence intervals, are transcribed from the clinical study report and were not recomputed.

Table S6. Subgroup AUROC at the 12-hour horizon.

Cohort	Input	Subgroup type	Subgroup	AUROC (95% CI)
Confirmatory trial	core	Overall	Overall	0.9103 (0.8716, 0.9490) [CSR]
Confirmatory trial	core	Sex	Male	0.9105 (0.8610, 0.9601) [CSR]
Confirmatory trial	core	Sex	Female	0.9197 (0.8580, 0.9813) [CSR]
Confirmatory trial	core	Age	Age < 65 years	0.9366 (0.8830, 0.9901) [CSR]
Confirmatory trial	core	Age	Age ≥ 65 years	0.9014 (0.8518, 0.9509) [CSR]
Confirmatory trial	core	ICU group	SICU family (incl. SICU2, CPICU)	0.9248 (0.8531, 0.9965) [CSR]
Confirmatory trial	core	ICU group	MICU	0.8807 (0.8046, 0.9567) [CSR]
Confirmatory trial	core	ICU group	EICU	0.9181 (0.8568, 0.9793) [CSR]
Confirmatory trial	extended	Overall	Overall	0.9428 (0.9102, 0.9753) [CSR]
Confirmatory trial	extended	Sex	Male	0.9501 (0.9140, 0.9863) [CSR]
Confirmatory trial	extended	Sex	Female	0.9261 (0.8560, 0.9962) [CSR]
Confirmatory trial	extended	Age	Age < 65 years	0.9522 (0.8974, 1.0000) [CSR]
Confirmatory trial	extended	Age	Age ≥ 65 years	0.9369 (0.8955, 0.9784) [CSR]
Confirmatory trial	extended	ICU group	SICU family (incl. SICU2, CPICU)	0.9640 (0.8992, 1.0000) [CSR]
Confirmatory trial	extended	ICU group	MICU	0.9297 (0.8735, 0.9858) [CSR]
Confirmatory trial	extended	ICU group	EICU	0.9432 (0.8907, 0.9957) [CSR]
Internal test set	core	Overall	Overall	0.9252 (0.9090–0.9392)
Internal test set	core	Sex	Male	0.9183 (0.8957–0.9372)
Internal test set	core	Sex	Female	0.9337 (0.9117–0.9533)
Internal test set	core	Age	Age < 65 years	0.9424 (0.9215–0.9590)
Internal test set	core	Age	Age ≥ 65 years	0.9112 (0.8871–0.9306)
Internal test set	core	ICU group	MICU	0.8953 (0.8610–0.9249)
Internal test set	core	ICU group	SICU family	0.9334 (0.9062–0.9542)
Internal test set	core	ICU group	EICU	0.9307 (0.9065–0.9522)
Internal test set	core	ICU group	Other	0.8787 (0.7913–0.9358)
Internal test set	core	ICU unit	CCU	0.9402 (0.8782–0.9781)
Internal test set	core	ICU unit	RICU	0.8436 (0.6812–0.9366)
Internal test set	core	ICU unit	SICU	0.9353 (0.8890–0.9660)
Internal test set	core	ICU unit	SICU2	0.9275 (0.8908–0.9549)
Internal test set	extended	Overall	Overall	0.9536 (0.9427–0.9629)
Internal test set	extended	Sex	Male	0.9533 (0.9385–0.9659)
Internal test set	extended	Sex	Female	0.9541 (0.9382–0.9678)
Internal test set	extended	Age	Age < 65 years	0.9679 (0.9561–0.9785)
Internal test set	extended	Age	Age ≥ 65 years	0.9408 (0.9230–0.9553)
Internal test set	extended	ICU group	MICU	0.9219 (0.8946–0.9444)
Internal test set	extended	ICU group	SICU family	0.9658 (0.9491–0.9785)
Internal test set	extended	ICU group	EICU	0.9465 (0.9225–0.9670)
Internal test set	extended	ICU group	Other	0.9132 (0.8401–0.9607)
Internal test set	extended	ICU unit	CCU	0.9167 (0.8119–0.9812)
Internal test set	extended	ICU unit	RICU	0.9116 (0.8043–0.9711)
Internal test set	extended	ICU unit	SICU	0.9701 (0.9456–0.9868)
Internal test set	extended	ICU unit	SICU2	0.9595 (0.9337–0.9775)
External cohort	core	Overall	Overall	0.9458 (0.9415–0.9500)
External cohort	core	Sex	Male	0.9426 (0.9369–0.9480)
External cohort	core	Sex	Female	0.9506 (0.9434–0.9571)
External cohort	core	Age	Age < 65 years	0.9531 (0.9472–0.9587)
External cohort	core	Age	Age ≥ 65 years	0.9381 (0.9315–0.9445)
External cohort	core	ICU group	MICU	0.9863 (0.9540–1.0000) †
External cohort	core	ICU group	SICU family	0.9578 (0.8935–0.9979) †
External cohort	core	ICU group	EICU	0.9444 (0.9357–0.9519)
External cohort	core	ICU group	Other	0.9461 (0.9406–0.9511)
External cohort	core	ICU unit	ICU 1	0.9005 (0.8814–0.9169)

Cohort	Input	Subgroup type	Subgroup	AUROC (95% CI)
External cohort	core	ICU unit	ICU 2	0.9426 (0.9304–0.9538)
External cohort	core	ICU unit	ICU 3	0.9612 (0.9528–0.9688)
External cohort	core	ICU unit	ICU 4	0.9511 (0.9430–0.9589)
External cohort	core	ICU unit	CSICU	0.6621 (0.6135–0.7038) †
External cohort	core	ICU unit	GSICU	0.9983 (0.9945–1.0000) †
External cohort	core	ICU unit	NSICU	0.9169 (0.8492–1.0000) †
External cohort	extended	Overall	Overall	0.9587 (0.9548–0.9626)
External cohort	extended	Sex	Male	0.9572 (0.9520–0.9620)
External cohort	extended	Sex	Female	0.9609 (0.9544–0.9669)
External cohort	extended	Age	Age < 65 years	0.9628 (0.9572–0.9679)
External cohort	extended	Age	Age ≥ 65 years	0.9541 (0.9482–0.9598)
External cohort	extended	ICU group	MICU	0.9925 (0.9782–0.9993) †
External cohort	extended	ICU group	SICU family	0.9909 (0.9762–0.9999) †
External cohort	extended	ICU group	EICU	0.9564 (0.9494–0.9635)
External cohort	extended	ICU group	Other	0.9592 (0.9545–0.9638)
External cohort	extended	ICU unit	ICU 1	0.9216 (0.9047–0.9382)
External cohort	extended	ICU unit	ICU 2	0.9552 (0.9423–0.9668)
External cohort	extended	ICU unit	ICU 3	0.9717 (0.9646–0.9783)
External cohort	extended	ICU unit	ICU 4	0.9625 (0.9552–0.9697)
External cohort	extended	ICU unit	CSICU	0.9579 (0.9457–0.9693) †
External cohort	extended	ICU unit	GSICU	0.9998 (0.9986–1.0000) †
External cohort	extended	ICU unit	NSICU	0.9728 (0.9501–1.0000) †

Levels of analysis are as in Table S5, with the internal test set per prediction point (Cohort column); estimates are not compared across cohorts. A dagger marks a stratum with fewer than ten positive admissions, listed for completeness and not interpreted. Single-unit groups (EICU, MICU) are not repeated as units. [CSR], transcribed from the clinical study report, not recomputed.

Table S7. SAPS-II scored as originally defined against the repeated form.

SAPS-II specification	n prediction points	n positive	AUROC (95% CI)	AUPRC (95% CI)
repeated form	4,367,775	69,079	0.8766 (0.8690–0.8845)	0.3497 (0.3248–0.3768)
frozen at 24 h (instrument as defined)	4,367,775	69,079	0.7313 (0.7192–0.7436)	0.0704 (0.0598–0.0872)

As originally defined the instrument is scored once from the first 24 hours; the repeated form rescores it at each prediction point. Both rows are at the 12-hour horizon, by prediction point, in the external cohort; the repeated form is the SAPS-II of Table 2.

Table S8. iCARS operating characteristics at the anchor-matched thresholds, the external cohort.

Input	Comparator anchor	Readout	iCARS threshold	Sensitivity (%)	Specificity (%)	PPV (%)	NNE	MACPD (8-h blocks/bed-day)	Lead time median (95% CI), h	Lead time IQR, h
extended	NEWS2 ≥7 (primary)	equal alarm rate (primary)	76	77.36 (76.04–78.59)	98.24 (98.08–98.38)	41.44 (38.87–43.89)	2.41 (2.28–2.57)	0.2666 (0.2552–0.2783)	11.42 (11.35–11.47)	6.41–11.78
extended	NEWS2 ≥7 (primary)	equal sensitivity (secondary)	100	41.62 (40.07–43.21)	99.92 (99.88–99.95)	89.33 (85.00–92.59)	1.12 (1.08–1.18)	0.0455 (0.0424–0.0488)	5.42 (4.87–6.00)	0.96–11.30
extended	SOFA ≥11 (primary)	equal alarm rate (primary)	66	80.47 (79.19–81.60)	97.34 (97.13–97.53)	32.74 (30.69–34.78)	3.05 (2.87–3.26)	0.3372 (0.3243–0.3503)	11.50 (11.43–11.52)	7.62–11.78
extended	SOFA ≥11 (primary)	equal sensitivity (secondary)	99	52.29 (50.67–53.81)	99.83 (99.78–99.86)	82.95 (78.78–86.35)	1.21 (1.16–1.27)	0.0693 (0.0651–0.0736)	7.97 (7.40–8.55)	1.93–11.57
extended	NEWS2 ≥6 (secondary)	equal alarm rate (primary)	48	84.85 (83.74–85.86)	95.04 (94.75–95.31)	21.58 (20.25–22.94)	4.63 (4.36–4.94)	0.4852 (0.4694–0.5015)	11.53 (11.50–11.55)	9.47–11.82
extended	NEWS2 ≥6 (secondary)	equal sensitivity (secondary)	97	59.89 (58.34–61.44)	99.69 (99.62–99.74)	75.43 (71.36–78.96)	1.33 (1.27–1.40)	0.0992 (0.0936–0.1051)	10.11 (9.47–10.62)	3.00–11.65
extended	SOFA ≥10 (secondary)	equal alarm rate (primary)	52	83.97 (82.81–85.03)	95.66 (95.38–95.90)	23.71 (22.27–25.20)	4.22 (3.97–4.49)	0.4481 (0.4329–0.4638)	11.53 (11.50–11.55)	9.17–11.80
extended	SOFA ≥10 (secondary)	equal sensitivity (secondary)	97	59.89 (58.34–61.44)	99.69 (99.62–99.74)	75.43 (71.36–78.96)	1.33 (1.27–1.40)	0.0992 (0.0936–0.1051)	10.11 (9.47–10.62)	3.00–11.65
core	NEWS2 ≥7 (primary)	equal alarm rate (primary)	62	76.04 (74.71–77.27)	96.90 (96.67–97.12)	28.85 (27.00–30.88)	3.47 (3.24–3.70)	0.2962 (0.2851–0.3079)	11.27 (11.23–11.33)	5.33–11.73
core	NEWS2 ≥7 (primary)	equal sensitivity (secondary)	98	42.10 (40.48–43.73)	99.88 (99.84–99.92)	85.55 (81.00–89.36)	1.17 (1.12–1.23)	0.0433 (0.0407–0.0460)	3.62 (3.13–4.02)	0.75–10.55
core	SOFA ≥11 (primary)	equal alarm rate (primary)	57	77.90 (76.63–79.08)	96.17 (95.91–96.41)	25.15 (23.55–26.92)	3.98 (3.71–4.25)	0.3401 (0.3281–0.3530)	11.32 (11.25–11.37)	6.12–11.75
core	SOFA ≥11 (primary)	equal sensitivity (secondary)	95	50.90 (49.27–52.49)	99.74 (99.67–99.79)	76.25 (71.76–80.33)	1.31 (1.24–1.39)	0.0660 (0.0622–0.0700)	5.63 (5.07–6.25)	1.03–11.33
core	NEWS2 ≥6 (secondary)	equal alarm rate (primary)	40	83.85 (82.71–84.85)	92.56 (92.17–92.92)	15.70 (14.73–16.76)	6.37 (5.97–6.79)	0.5225 (0.5073–0.5393)	11.45 (11.42–11.50)	8.33–11.77
core	NEWS2 ≥6 (secondary)	equal sensitivity (secondary)	90	58.36 (56.76–59.94)	99.47 (99.37–99.55)	64.47 (60.36–68.36)	1.55 (1.46–1.66)	0.0993 (0.0941–0.1049)	7.82 (7.22–8.38)	1.80–11.55
core	SOFA ≥10 (secondary)	equal alarm rate (primary)	46	81.79 (80.57–82.86)	94.08 (93.74–94.41)	18.61 (17.43–19.88)	5.37 (5.03–5.74)	0.4497 (0.4357–0.4653)	11.42 (11.37–11.48)	7.71–11.77
core	SOFA ≥10 (secondary)	equal sensitivity (secondary)	90	58.36 (56.76–59.94)	99.47 (99.37–99.55)	64.47 (60.36–68.36)	1.55 (1.46–1.66)	0.0993 (0.0941–0.1049)	7.82 (7.22–8.38)	1.80–11.55

The thresholds are those of Table S1, on the scale of Section 2.3, and are computed reference points rather than operating thresholds we propose; comparator operating characteristics are in Table S2. Sensitivity, specificity, PPV and NNE are prediction-point quantities computed on the discrimination set, whereas MACPD covers the whole observation period (Table S12). Lead time

is defined once per admission, from the first above-threshold prediction to the event, conditional on an alarm having occurred and right-truncated by the 12-hour label window; the interquartile range is a descriptive spread rather than an interval estimate. NNE, number needed to evaluate, the reciprocal of the positive predictive value. Parenthetical ranges are 95% confidence intervals as in Table S1.

Table S9. Calibration in the confirmatory trial, the internal test set and the external cohort.

Cohort	Input	n	n positive	Mean predicted value (%)	Observed (%)	O:E (95% CI)	Brier (95% CI)	ECE (95% CI)	Calibration slope (95% CI)	Calibration intercept (95% CI)
Confirmatory trial	extended	214	107	49.10	50.00	1.02	0.0864 [CSR]	0.0321 (0.0249–0.0849)	n/a	n/a
Confirmatory trial	core	214	107	55.86	50.00	0.90	0.1270 [CSR]	0.0664 (0.0571–0.1358)	n/a	n/a
Internal test set	extended	820,495	13,516	1.71	1.65	0.96 (0.86–1.07)	0.0088 (0.0076–0.0102)	0.0027 (0.0013–0.0042)	0.8175 (0.7499–0.8997)	-0.0890 (-0.3663 to 0.1513)
Internal test set	core	743,893	12,458	1.69	1.67	0.99 (0.88–1.11)	0.0105 (0.0091–0.0120)	0.0015 (0.0008–0.0033)	0.9055 (0.8311–0.9899)	-0.0131 (-0.2078 to 0.1732)
External cohort	extended	4,367,775	69,079	1.48	1.58	1.07 (1.02–1.12)	0.0076 (0.0072–0.0082)	0.0010 (0.0006–0.0017)	0.9031 (0.8539–0.9454)	0.1587 (0.0433–0.2698)
External cohort	core	3,754,387	61,073	1.25	1.63	1.30 (1.24–1.36)	0.0090 (0.0086–0.0095)	0.0038 (0.0031–0.0045)	1.0235 (0.9784–1.0663)	0.4860 (0.4011–0.5681)

In the internal test set and the external cohort the predicted value is the absolute 12-hour risk of Section 2.3, with its calibration estimated on the development data and applied unchanged; statistics are at the prediction-point level in the discrimination set and unweighted, so the observed column is the cohort's own event rate. In the confirmatory trial, whose 1:1 design fixes the observed event fraction at 50%, the predicted value is the device's output and statistics are at the subject level. n is those units in the discrimination set, n positive those inside the label window; O:E is the observed over the mean predicted value. ECE, expected calibration error (Section 2.8), biased upward in finite samples, so its interval is not centred on the point estimate; the trial's eight-bin value is not comparable with the other cohorts. Calibration slope is the coefficient of a logistic regression of the outcome on the logit of the predicted value, and the calibration intercept that of the same regression with the slope fixed at 1. On the extended input, scores of 50, 66, 76, 90 and 99 correspond to absolute 12-hour risks of 2.0%, 3.7%, 5.8%, 14.5% and 63.8% (core input: 2.1%, 3.9%, 6.1%, 15.3% and 65.4%). [CSR], transcribed from the clinical study report, not recomputed. n/a, not computed for the matched trial sample. Confidence intervals are as in Section 2.8.

Table S10. Machine-learning benchmark models trained on the development set, fitted without hyperparameter tuning.

(a) Extended input.

Cohort	Unit	Model	n evaluated	n positive	AUROC (95% CI)	p vs iCARS
Internal test set	8-hour block	Logistic regression	60,786	941	0.8748 (0.8542–0.8935)	≤0.001
Internal test set	8-hour block	Random forest	60,786	941	0.8614 (0.8393–0.8817)	≤0.001
Internal test set	8-hour block	Gradient boosting	60,786	941	0.8804 (0.8617–0.8981)	≤0.001
Internal test set	8-hour block	iCARS (reference)	60,786	941	0.9416 (0.9281–0.9537)	-
Internal test set	prediction point	Logistic regression	820,495	13,516	0.8994 (0.8820–0.9150)	≤0.001
Internal test set	prediction point	Random forest	820,495	13,516	0.8808 (0.8615–0.8983)	≤0.001
Internal test set	prediction point	Gradient boosting	820,495	13,516	0.8950 (0.8766–0.9119)	≤0.001
Internal test set	prediction point	iCARS (reference)	820,495	13,516	0.9536 (0.9427–0.9629)	-
External cohort	8-hour block	Logistic regression	384,216	6,129	0.9034 (0.8969–0.9095)	≤0.001
External cohort	8-hour block	Random forest	384,216	6,129	0.8812 (0.8737–0.8883)	≤0.001
External cohort	8-hour block	Gradient boosting	384,216	6,129	0.9000 (0.8930–0.9062)	≤0.001
External cohort	8-hour block	iCARS (reference)	384,216	6,129	0.9543 (0.9494–0.9588)	-
External cohort	prediction point	Logistic regression	4,367,775	69,079	0.9009 (0.8945–0.9070)	≤0.001
External cohort	prediction point	Random forest	4,367,775	69,079	0.8680 (0.8607–0.8755)	≤0.001
External cohort	prediction point	Gradient boosting	4,367,775	69,079	0.8936 (0.8869–0.9000)	≤0.001
External cohort	prediction point	iCARS (reference)	4,367,775	69,079	0.9587 (0.9548–0.9626)	-

(b) Core input.

Cohort	Unit	Model	n evaluated	n positive	AUROC (95% CI)	p vs iCARS
Internal test set	8-hour block	Logistic regression	60,376	940	0.8465 (0.8254–0.8661)	≤0.001
Internal test set	8-hour block	Random forest	60,376	940	0.8218 (0.8001–0.8441)	≤0.001
Internal test set	8-hour block	Gradient boosting	60,376	940	0.8604 (0.8409–0.8795)	≤0.001
Internal test set	8-hour block	iCARS (reference)	60,376	940	0.9079 (0.8898–0.9251)	-
Internal test set	prediction point	Logistic regression	743,893	12,458	0.8637 (0.8452–0.8812)	≤0.001
Internal test set	prediction point	Random forest	743,893	12,458	0.8280 (0.8051–0.8489)	≤0.001
Internal test set	prediction point	Gradient boosting	743,893	12,458	0.8799 (0.8603–0.8972)	≤0.001
Internal test set	prediction point	iCARS (reference)	743,893	12,458	0.9252 (0.9090–0.9392)	-
External cohort	8-hour block	Logistic regression	380,532	6,099	0.8872 (0.8805–0.8934)	≤0.001
External cohort	8-hour block	Random forest	380,532	6,099	0.8588 (0.8515–0.8661)	≤0.001
External cohort	8-hour block	Gradient boosting	380,532	6,099	0.8895 (0.8830–0.8960)	≤0.001
External cohort	8-hour block	iCARS (reference)	380,532	6,099	0.9373 (0.9318–0.9425)	-
External cohort	prediction point	Logistic regression	3,754,387	61,073	0.8827 (0.8751–0.8900)	≤0.001
External cohort	prediction point	Random forest	3,754,387	61,073	0.8386 (0.8317–0.8460)	≤0.001
External cohort	prediction point	Gradient boosting	3,754,387	61,073	0.8939 (0.8882–0.8994)	≤0.001
External cohort	prediction point	iCARS (reference)	3,754,387	61,073	0.9458 (0.9415–0.9500)	-

Each cohort-and-unit block ends with the iCARS reference row against which that block's p value is computed, by paired bootstrap on common evaluation units; in panel (b) every model is evaluated on the units the core input scores. Each 8-hour block takes its highest score and is positive if it contains a positive prediction point. The evaluation set follows the convention of Tables S5 and S6, so the prediction-point iCARS rows are the values reported there and in Table 2.

Table S11. Plausible ranges applied to the reported input distributions.

Variable	Lower bound	Upper bound
Respiratory rate, /min	0	80
Heart rate, /min	0	300
Systolic blood pressure, mmHg	20	300
Diastolic blood pressure, mmHg	5	200
Body temperature, °C	25	45
SpO2, %	10	100
GCS — eye	1	4
GCS — verbal	1	5
GCS — motor	1	6
ALT, U/L	0	5000
AST, U/L	0	10,000
Albumin, g/dL	0	5.5
Blood urea nitrogen, mg/dL	1	300
Bilirubin, mg/dL	0	80
C-reactive protein, mg/dL	0	300
Chloride, mmol/L	70	135
Creatinine, mg/dL	0.05	30
Glucose, mg/dL	0	600
Hemoglobin, g/dL	1	25
Prothrombin time, INR	0	8
Platelet count, 10^3/μL	0	2000
Potassium, mmol/L	0.5	12
Sodium, mmol/L	90	200
White cell count, 10^3/μL	0	500
aPTT, s	0	300
Bicarbonate, mmol/L	1	60
Lactate, mmol/L	0	30
pCO2, mmHg	5	200
pH	6.5	8
pO2, mmHg	10	760

These ranges were used only to summarise the input distributions in Table 1; values outside them were excluded from those summaries rather than truncated. The same ranges were applied to all three cohorts. No confirmatory-trial observation fell outside a range; externally 415,799 did, 413,355 of them a Glasgow Coma Scale verbal component recorded as 0 where the eye and motor components were scored; these unassessable verbal responses entered the device as missing (Section 2.3).

Table S12. Alarm burden under the two row-set conventions, the external cohort.

Alarm set	Comparator anchor	Readout	Comparator sensitivity (%)	Comparator MACPD	Matched iCARS threshold (extended input)	iCARS sensitivity (%)	iCARS MACPD	MACPD ratio, fold
Full observation period (reported)	NEWS2 ≥7	equal alarm rate (primary)	40.19	0.757 (0.740–0.773)	76	77.36	0.2666 (0.2552–0.2783)	2.84× (2.73–2.95)
Full observation period (reported)	NEWS2 ≥7	equal sensitivity (secondary)	40.19	0.757 (0.740–0.773)	100	41.62	0.0455 (0.0424–0.0488)	16.64× (15.53–17.79)
Full observation period (reported)	SOFA ≥11	equal alarm rate (primary)	49.81	0.341 (0.324–0.358)	66	80.47	0.3372 (0.3243–0.3503)	1.01× (0.96–1.06)
Full observation period (reported)	SOFA ≥11	equal sensitivity (secondary)	49.81	0.341 (0.324–0.358)	99	52.29	0.0693 (0.0651–0.0736)	4.93× (4.60–5.28)
Discrimination set	NEWS2 ≥7	equal alarm rate (primary)	40.19	0.650 (0.634–0.667)	76	77.36	0.1531 (0.1467–0.1606)	4.24× (4.05–4.43)
Discrimination set	NEWS2 ≥7	equal sensitivity (secondary)	40.19	0.650 (0.634–0.667)	100	41.62	0.0305 (0.0289–0.0323)	21.30× (20.03–22.57)
Discrimination set	SOFA ≥11	equal alarm rate (primary)	49.81	0.214 (0.202–0.228)	66	80.47	0.2038 (0.1960–0.2129)	1.05× (0.98–1.12)
Discrimination set	SOFA ≥11	equal sensitivity (secondary)	49.81	0.214 (0.202–0.228)	99	52.29	0.0407 (0.0385–0.0431)	5.27× (4.86–5.71)

The discrimination set omits the points earlier than the label window in event admissions (Section 2.5), 20.09% of prediction points and 17.68% of bed-days. Sensitivity is identical under either convention, which moves only specificity and the areas under the curves: counted as negatives, specificity would fall from 97.34% to 92.83% at iCARS 66 and from 95.18% to 90.90% at SOFA ≥11 (Table S14). Applying the discrimination convention to the alarm set as well raises every MACPD ratio in the table, so the main-text values are the conservative ones. Parenthetical ranges are 95% confidence intervals as in Table S1, from which the sensitivities are taken.

Table S13. Discrimination with the positive set restricted by event type, the internal test set and the external cohort.

Cohort	Positive set	n positive admissions	iCARS, extended input	iCARS, core input	NEWS2	SOFA	Difference vs NEWS2	Difference vs SOFA
Internal test set	Both event types (reference)	437	0.9536 (0.9427–0.9629)	0.9252 (0.9090–0.9392)	0.7404 (0.7184–0.7607)	0.8000 (0.7733–0.8252)	+0.2140 (0.1943–0.2355)	+0.1536 (0.1288–0.1792)
Internal test set	Cardiac arrest with CPR	170	0.9031 (0.8775–0.9271)	0.8609 (0.8268–0.8936)	0.6967 (0.6602–0.7314)	0.7471 (0.6971–0.7919)	+0.2081 (0.1773–0.2416)	+0.1560 (0.1138–0.2073)
Internal test set	Death without CPR	267	0.9804 (0.9739–0.9861)	0.9590 (0.9487–0.9686)	0.7634 (0.7388–0.7877)	0.8281 (0.7996–0.8551)	+0.2171 (0.1925–0.2420)	+0.1523 (0.1263–0.1794)
External cohort	Both event types (reference)	2,782 (2,770)	0.9587 (0.9548–0.9626)	0.9458 (0.9415–0.9500)	0.8232 (0.8169–0.8295)	0.8583 (0.8498–0.8665)	+0.1355 (0.1301–0.1412)	+0.1004 (0.0932–0.1079)
External cohort	Cardiac arrest with CPR	653 (647)	0.8697 (0.8538–0.8854)	0.8425 (0.8264–0.8591)	0.7157 (0.6987–0.7319)	0.7424 (0.7205–0.7631)	+0.1540 (0.1402–0.1678)	+0.1273 (0.1110–0.1467)
External cohort	Death without CPR	2,129 (2,123)	0.9799 (0.9775–0.9823)	0.9690 (0.9658–0.9719)	0.8489 (0.8427–0.8552)	0.8860 (0.8777–0.8944)	+0.1311 (0.1252–0.1369)	+0.0940 (0.0857–0.1023)

Within each cohort the negative points are held exactly fixed, so this restricts the positive set rather than relabelling the outcome. External events are classified as in Section 2.4: cardiac arrest with CPR comprises admissions with a CPR record and those whose cardiac-arrest record was not followed by death within 24 hours of ICU discharge. n positive admissions counts admissions contributing at least one positive prediction point, with the extended and, in parentheses where it differs, the core input. Each comparator is evaluated on its own scored points, as in Table 2; each difference is iCARS with the extended input minus the comparator, on the points both score. Every difference, with either input, had $p \leq 0.001$ by paired bootstrap. Parenthetical ranges are 95% confidence intervals as in Table S1.

Table S14. Discrimination, operating characteristics and timing with the pre-window points of event admissions counted as negatives, the external cohort.

(a) Discrimination.

Score	AUROC, discrimination set	AUROC, pre-window points counted as negatives
iCARS, extended input	0.9587 (0.9548–0.9626)	0.9375 (0.9331–0.9420)
iCARS, core input	0.9458 (0.9415–0.9500)	0.9270 (0.9224–0.9316)
NEWS2	0.8232 (0.8169–0.8295)	0.8039 (0.7977–0.8102)
SOFA	0.8583 (0.8498–0.8665)	0.8191 (0.8107–0.8272)

(b) Operating characteristics at the alarm-burden-matched thresholds and the comparator anchors.

Score and threshold	Sensitivity (%)	Specificity (%), discrimination set	Specificity (%), pre-window points as negatives	PPV (%), discrimination set	PPV (%), pre-window points as negatives	NNE, discrimination set	NNE, pre-window points as negatives
iCARS, extended input, 66 (alarm burden of SOFA ≥11)	80.47 (79.19–81.60)	97.34 (97.13–97.53)	92.83 (92.42–93.21)	32.74 (30.69–34.78)	12.56 (11.93–13.24)	3.05 (2.87–3.26)	7.96 (7.55–8.38)
SOFA ≥11	49.81 (47.91–51.72)	95.18 (94.77–95.56)	90.90 (90.37–91.43)	14.24 (13.02–15.58)	6.55 (6.17–6.97)	7.02 (6.42–7.68)	15.27 (14.34–16.22)
iCARS, extended input, 76 (alarm burden of NEWS2 ≥7)	77.36 (76.04–78.59)	98.24 (98.08–98.38)	94.50 (94.17–94.83)	41.44 (38.87–43.89)	15.27 (14.47–16.13)	2.41 (2.28–2.57)	6.55 (6.20–6.91)
NEWS2 ≥7	40.19 (38.99–41.32)	95.65 (95.47–95.81)	94.02 (93.81–94.21)	12.92 (12.14–13.73)	7.92 (7.53–8.31)	7.74 (7.28–8.24)	12.63 (12.04–13.28)
iCARS, core input, 57 (alarm burden of SOFA ≥11)	77.90 (76.63–79.08)	96.17 (95.91–96.41)	92.36 (91.96–92.73)	25.15 (23.55–26.92)	11.86 (11.23–12.48)	3.98 (3.71–4.25)	8.43 (8.01–8.91)
iCARS, core input, 62 (alarm burden of NEWS2 ≥7)	76.04 (74.71–77.27)	96.90 (96.67–97.12)	93.46 (93.10–93.82)	28.85 (27.00–30.88)	13.30 (12.59–14.03)	3.47 (3.24–3.70)	7.52 (7.13–7.94)

(c) Status at window opening and whole-stay lead time.

Score and threshold	Event admissions, n	Above threshold at window opening, % of all	Above threshold at window opening, % of those with an earlier prediction	Whole-stay lead time, median (95% CI), h	Whole-stay lead time, IQR, h
iCARS, extended input, 66 (alarm burden of SOFA ≥11)	2,782	58.30 (56.41–60.16)	67.67 (65.68–69.55)	80.84 (75.05–88.80)	20.85–262.50
SOFA ≥11	2,781	39.66 (37.87–41.59)	46.02 (44.05–48.10)	84.80 (75.37–92.62)	24.80–264.02
iCARS, extended input, 76 (alarm burden of NEWS2 ≥7)	2,782	53.81 (51.87–55.67)	62.45 (60.40–64.37)	72.42 (66.12–79.13)	18.30–248.54

Score and threshold	Event admissions, n	Above threshold at window opening, % of all	Above threshold at window opening, % of those with an earlier prediction	Whole-stay lead time, median (95% CI), h	Whole-stay lead time, IQR, h
NEWS2 ≥7	2,781	31.14 (29.41–32.89)	36.14 (34.20–38.05)	97.77 (89.37–107.42)	24.83–305.25
iCARS, core input, 57 (alarm burden of SOFA ≥11)	2,770	51.30 (49.44–53.19)	59.58 (57.56–61.61)	81.25 (75.53–88.30)	20.22–259.53
iCARS, core input, 62 (alarm burden of NEWS2 ≥7)	2,770	48.88 (46.95–50.79)	56.77 (54.69–58.78)	77.37 (71.48–83.92)	19.00–254.17

The discrimination set (Section 2.5) omits the prediction points earlier than the 12-hour window in event admissions; here they are also shown counted as negatives, as a deployed device would alarm on them. Sensitivity is the same under both conventions. The iCARS thresholds are those matched on alarm burden to SOFA ≥11 and NEWS2 ≥7 (Table S1); the matching uses the whole observation period, so the convention does not change it. In panel (c), an event admission is above threshold at window opening if its last prediction before the 12-hour window reached the threshold, given as a share of all event admissions and of those with a prediction before the window, and the whole-stay lead time runs from the first above-threshold prediction in the admission to the event. For each score, event admissions are those with a positive point in its own discrimination set. NNE, number needed to evaluate. Parenthetical ranges are 95% confidence intervals as in Table S1; the interquartile range is a descriptive spread rather than an interval estimate.

Table S15. Discrimination and sensitivity by interval before the event.

(a) AUROC.

Cohort	Score	0–3 h	3–6 h	6–9 h	9–12 h
Confirmatory trial	iCARS, extended input	0.9383 (0.9013–0.9694)	0.9252 (0.8861–0.9577)	0.8989 (0.8536–0.9380)	0.9020 (0.8588–0.9414)
Confirmatory trial	iCARS, core input	0.9320 (0.8965–0.9621)	0.8906 (0.8451–0.9324)	0.8591 (0.8081–0.9047)	0.7903 (0.7320–0.8496)
Internal test set	iCARS, extended input	0.9594 (0.9482–0.9693)	0.9537 (0.9407–0.9648)	0.9489 (0.9353–0.9603)	0.9463 (0.9329–0.9573)
Internal test set	iCARS, core input	0.9461 (0.9333–0.9574)	0.9242 (0.9050–0.9409)	0.9018 (0.8688–0.9275)	0.9056 (0.8861–0.9223)
Internal test set	NEWS2	0.7521 (0.7226–0.7803)	0.7333 (0.6962–0.7701)	0.7435 (0.7148–0.7699)	0.7207 (0.6879–0.7532)
Internal test set	SOFA	0.7980 (0.7615–0.8303)	0.8036 (0.7747–0.8302)	0.7975 (0.7659–0.8270)	0.8023 (0.7736–0.8281)
External cohort	iCARS, extended input	0.9744 (0.9712–0.9774)	0.9540 (0.9493–0.9589)	0.9489 (0.9438–0.9540)	0.9451 (0.9398–0.9500)
External cohort	iCARS, core input	0.9709 (0.9678–0.9739)	0.9384 (0.9327–0.9438)	0.9299 (0.9240–0.9356)	0.9191 (0.9125–0.9251)
External cohort	NEWS2	0.8412 (0.8328–0.8491)	0.8225 (0.8143–0.8306)	0.8140 (0.8057–0.8218)	0.8005 (0.7923–0.8084)
External cohort	SOFA	0.8630 (0.8513–0.8736)	0.8540 (0.8441–0.8630)	0.8579 (0.8487–0.8669)	0.8548 (0.8459–0.8638)

(b) Sensitivity (%), the external cohort.

Score and threshold	0–3 h	3–6 h	6–9 h	9–12 h
iCARS, extended input, 66	88.51 (87.47–89.57)	78.30 (76.70–79.91)	75.82 (74.17–77.45)	72.80 (70.99–74.49)
iCARS, extended input, 76	86.66 (85.50–87.82)	74.97 (73.23–76.75)	71.96 (70.20–73.75)	68.40 (66.53–70.17)
iCARS, core input, 57	88.19 (87.12–89.19)	74.76 (73.09–76.48)	71.18 (69.35–72.98)	67.26 (65.41–69.13)
iCARS, core input, 62	86.97 (85.84–88.03)	72.88 (71.16–74.65)	68.73 (66.85–70.58)	64.72 (62.78–66.66)
SOFA ≥11	50.86 (47.84–53.65)	49.24 (46.93–51.54)	49.72 (47.44–51.89)	48.55 (46.49–50.65)
NEWS2 ≥7	39.10 (37.24–40.82)	43.32 (41.65–45.02)	41.10 (39.46–42.74)	37.88 (36.38–39.51)

Each interval keeps the negative points of the 12-hour analysis and admits as positive only the prediction points in that interval, so the columns differ only in their positive sets; the 12-hour values are in Table 2, Table S1 and Table S13. Confirmatory-trial values are at the subject level, from each subject's maximum score inside the interval of its case-report-form window, computed on the pipeline scores that reproduce the clinical study report; two cases had no prediction 9–12 hours before the event. Sensitivity is at the iCARS thresholds matched on alarm burden (Table S1) and at the comparator anchors. Parenthetical ranges are 95% confidence intervals from patient-level (trial: subject-level) clustered bootstrap with 2,000 resamples.

Table S16. External event records and discrimination for arrests with CPR before and after the 2018 legislation on life-sustaining treatment decisions.

Period (ICU admission)	ICU admissions, n	CPR or cardiac-arrest record, % of admissions	CPR record, % of admissions	Arrests with CPR, n / positive admissions (%)	AUROC, arrests with CPR, extended input	AUROC, arrests with CPR, core input	AUROC, all events, extended input	AUROC, all events, core input
Before 4 February 2018	18,519	7.57	1.59	336 / 1,598 (21.03)	0.8725 (0.8498–0.8940)	0.8463 (0.8202–0.8699)	0.9634 (0.9586–0.9680)	0.9518 (0.9461–0.9568)
From 4 February 2018	11,968	8.76	2.16	317 / 1,184 (26.77)	0.8677 (0.8442–0.8898)	0.8379 (0.8146–0.8608)	0.9522 (0.9452–0.9587)	0.9370 (0.9296–0.9438)
All (2010–2021)	30,487	8.03	1.81	653 / 2,782 (23.47)	0.8697 (0.8538–0.8854)	0.8425 (0.8264–0.8591)	0.9587 (0.9548–0.9626)	0.9458 (0.9415–0.9500)

Periods are by ICU admission, before and from 4 February 2018, when the Korean act on life-sustaining treatment decisions took effect. The PNUYH structured record holds a CPR record and a cardiac-arrest record, each with a time only (Section 2.4); of the 2,448 event admissions with either record, 1,897 had only a cardiac-arrest record, 94.9% of those patients died in hospital, and the record typically fell about an hour before ICU discharge. Arrests with CPR are as classified in Section 2.4, and positive admissions are those with a prediction point in the 12-hour window (extended input). AUROC is at the prediction-point level with the non-event points held fixed, as in Table S13. Parenthetical ranges are 95% confidence intervals from patient-level clustered bootstrap with 2,000 resamples within each period.
